# Class Enumeration and Parameter Recovery of Growth Mixture Modeling and Second-Order Growth Mixture Modeling in the Presence of Measurement Noninvariance between Latent Classes

**DOI:** 10.3389/fpsyg.2017.01499

**Published:** 2017-09-05

**Authors:** Eun Sook Kim, Yan Wang

**Affiliations:** Department of Educational and Psychological Studies, University of South Florida Tampa, FL, United States

**Keywords:** growth mixture modeling, second-order growth mixture modeling, measurement invariance, latent class, class enumeration

## Abstract

Population heterogeneity in growth trajectories can be detected with growth mixture modeling (GMM). It is common that researchers compute composite scores of repeated measures and use them as multiple indicators of growth factors (baseline performance and growth) assuming measurement invariance between latent classes. Considering that the assumption of measurement invariance does not always hold, we investigate the impact of measurement noninvariance on class enumeration and parameter recovery in GMM through a Monte Carlo simulation study (Study 1). In Study 2, we examine the class enumeration and parameter recovery of the second-order growth mixture modeling (SOGMM) that incorporates measurement models at the first order level. Thus, SOGMM estimates growth trajectory parameters with reliable sources of variance, that is, common factor variance of repeated measures and allows heterogeneity in measurement parameters between latent classes. The class enumeration rates are examined with information criteria such as AIC, BIC, sample-size adjusted BIC, and hierarchical BIC under various simulation conditions. The results of Study 1 showed that the parameter estimates of baseline performance and growth factor means were biased to the degree of measurement noninvariance even when the correct number of latent classes was extracted. In Study 2, the class enumeration accuracy of SOGMM depended on information criteria, class separation, and sample size. The estimates of baseline performance and growth factor mean differences between classes were generally unbiased but the size of measurement noninvariance was underestimated. Overall, SOGMM is advantageous in that it yields unbiased estimates of growth trajectory parameters and more accurate class enumeration compared to GMM by incorporating measurement models.

## Introduction

In educational and psychological research the change or growth in temporal outcomes (e.g., alcohol use, depression, antisocial behavior, reading skills over time) is one of the major research questions (e.g., Muthén et al., 2000; Li et al., 2001; Miner and Clarke-Stewart, 2008). Given that the growth over time is likely variant across units of analysis (e.g., children), researchers are often interested in clustering in terms of the pattern or trend of growth. To investigate potential unobserved groups or latent classes in growth trajectories growth mixture modeling (GMM) is often used. For example, using GMM Baams et al. (2014) found resilients, undercontrollers, and overcontrollers in personality types; Hill et al. (2017) identified mild, increasing, elevated, and decreasing trajectories of depressive symptoms; and Oshri et al. (2017) observed declining, ascending, and stable high self-esteem.

Like many statistical methods, GMM is based on statistical assumptions. It is generally expected that the results of a statistical method are compromised to the extent to which statistical assumptions of the method are violated. One of the major assumptions of GMM is measurement invariance of longitudinal outcomes across latent classes that emerge from the data (Grimm and Ram, 2009). However, it is not known how the violation of the measurement invariance assumption impacts the performance of GMM. Thus, this study investigated the behaviors of GMM under the violation of measurement invariance across latent classes. Furthermore, we proposed the second-order growth mixture modeling (SOGMM) that allows modeling and testing measurement invariance explicitly across latent classes in the growth mixture analysis.

In the following section we first introduced latent growth modeling (LGM) that is a basic building block of second-order LGM and, next, discussed the advantages of second-order LGM addressing measurement invariance issues between observed groups in LGM. Then, we shift the focus to GMM for unobserved groups in growth trajectories and its extension to second-order GMM raising the issues of measurement noninvariance across latent classes.

### Latent growth modeling and second-order latent growth modeling

When researchers are interested in changes of individuals over time (e.g., changes in social role functioning over time in developmental psychology), LGM is often employed. LGM is appropriate to address research questions about the (a) average baseline performance, (b) average growth trajectories, (c) variability in baseline performance, and (d) variability in growth trajectories across individuals. That is, in addition to estimating the mean level of initial performance and growth, it allows those growth parameters to randomly vary across individuals (i.e., random effects). For example, in a study investigating the development of depressive symptoms of 7th graders over 3 years, the average depressive symptoms at grade 7 and the average growth rate of depressive symptoms over 3 years can be estimated with LGM. In addition, psychologists will be informed of how much variability exists among adolescents in terms of their initial depressive symptoms and growth rates.

**Figure 1:**
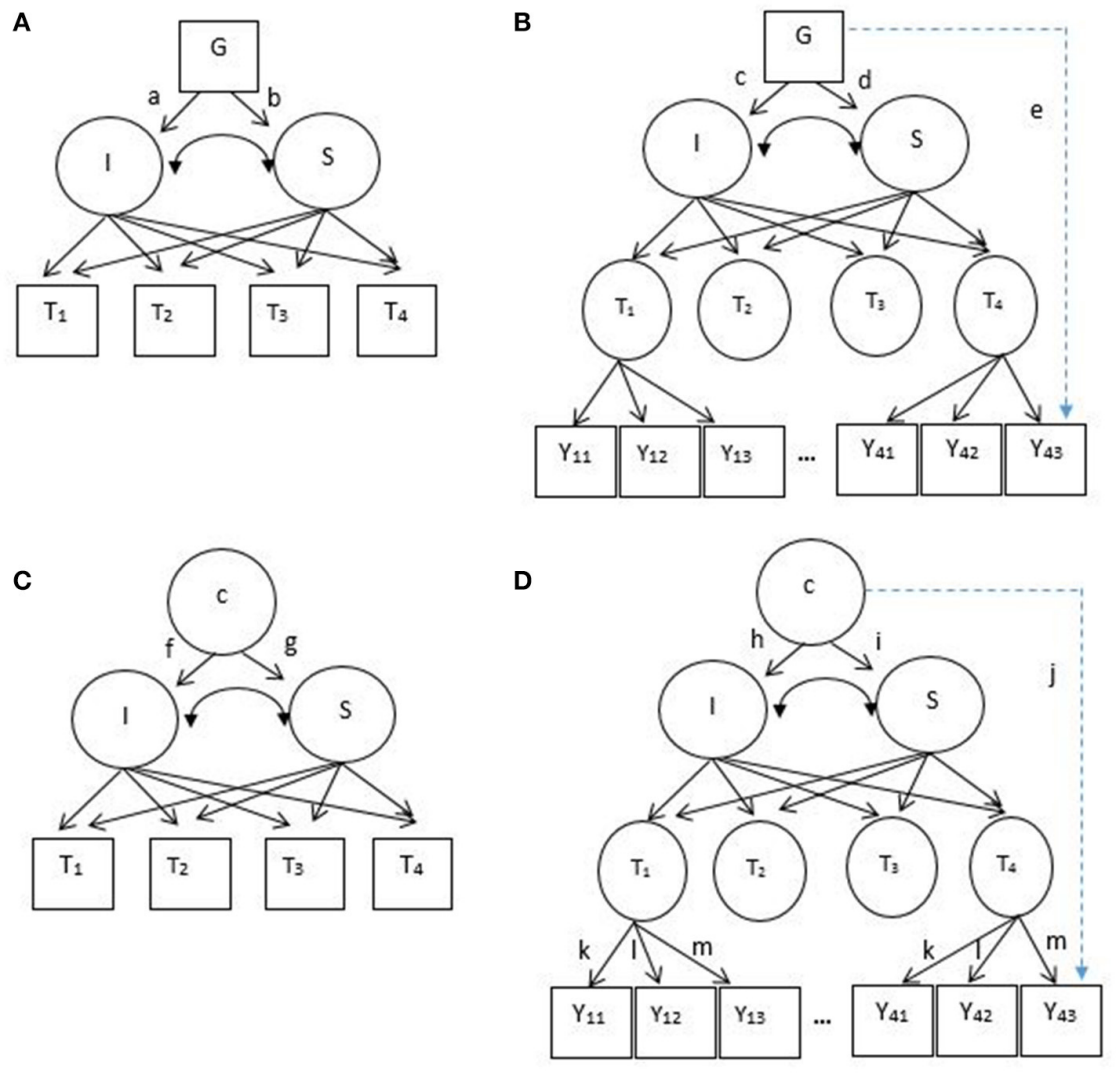
**(A)** Latent growth model, LGM **(B)** second-order latent growth model, SOLGM **(C)** growth mixture model, GMM **(D)** second-order growth mixture model, SOGMM. I = continuous latent intercept, S, continuous latent slope; c, unobserved categorical variable or latent classes; G, observed covariate (e.g., gender). T_1_–T_4_ are observed longitudinal outcome variables (squares) in LGM and GMM, but latent factors (circles) in SOLGM and SOGMM. Y_11_–Y_43_ are observed items of latent factors, T_1_–T_4_. Note that Y_21_–Y_33_ are not shown due to a limited space. Paths a–d represent covariate effects on the intercept and slope factors (or group-specific effects if a covariate is categorical). Paths f–i represent class-specific effects on the intercept and slope factors. Path e (a dotted line) represent a covariate effect on an item (measurement noninvariance in terms of a covariate). Path j (a dotted line) represent a class-specific effect on an item (measurement noninvariance between latent classes).

In LGM, researchers can also incorporate covariates to explain the variability in the baseline scores and growth rates of depressive symptoms. For example, when gender difference is expected in the development of depressive symptoms among adolescents, this effect can be modeled and tested in LGM as shown in Figure 1A: gender differences in baseline depressive symptoms and growth trajectories (paths a and b, respectively). In estimating these gender differences, LGM assumes measurement invariance of depressive symptoms between boys and girls. In other words, it is assumed that boys and girls respond to the items of a depressive symptoms checklist in the same manner.

However, this assumption of measurement invariance between boys and girls can be violated, which is illustrated in Figure 1B. In this figure, gender differences are present not only in the initial performance and growth rates of adolescents but also in their responses to an item that measures depressive symptoms (denoted by path e). When measurement invariance between boys and girls is violated, it is well-known that the mean comparison between them is not legitimate. Generally, scalar measurement invariance (i.e., invariance of factor structure, factor loadings, and intercepts of a measurement model) is required for meaningful mean comparisons between groups (Millsap and Kwok, 2004). Specifically in the context of LGM, Kim and Willson (2014a) investigated the impact of measurement noninvariance between groups on the performance of LGM and demonstrated that intercept noninvariance was directly associated with bias and Type I error inflation on the group effect on baseline performance (path a in Figure 1A) whereas factor loading noninvariance was associated with bias and Type I error inflation on the growth rate (path b in Figure 1A). To explicitly test measurement invariance in LGM, they recommended the second-order LGM (SOLGM).

As shown in Figure 1B, SOLGM includes measurement models of longitudinal outcome variables as the first-order part (McArdle, 1988; Meredith and Tisak, 1990). In LGM, the temporal outcomes are observed variables measured repeatedly over a period of time (squares denoted by T_1_–T_4_ in Figure 1A). When multiple items are used to measure the outcome (e.g., depressive symptoms), it is a common practice to use composite scores of the items (Leite, 2007). When composite scores are created, all items in a measure are equally weighted regardless of their relation to the latent factor measured. On the other hand, in SOLGM the temporal outcomes are latent factors that are measured by multiple items (circles denoted by T_1_–T_4_ in Figure 1B). Thus, the relations of items to the factors are explicitly modeled with different weights (i.e., factor loadings). Because unique factor variance or error variance is taken out, growth parameters are estimated with reliable sources of variance (common factor variance; McArdle, 1988; Grimm and Ram, 2009). In addition, measurement invariance (both longitudinal invariance and group invariance) can be examined with SOLGM (Kim and Willson, 2014b).

### Growth mixture modeling

Although, LGM is very useful providing information of the average initial performance and growth trajectory, it is generally assumed that all individuals are from a single population and thus the same growth pattern is applied to all individuals (Muthén, 2004; Frankfurt et al., 2016). However, it is often observed in social sciences that individuals change over time and those changes are not homogeneous across individuals. For example, the development patterns of depressive symptoms could be different among adolescents. Not to mention potential heterogeneity in baseline depressive symptoms, some may exhibit stably low or high symptoms over time, some may show steadily increasing trend, and others may experience exponential change. GMM allows researchers and practitioners to investigate the heterogeneity of growth patterns across individuals by combining the latent class approach or mixture modeling to LGM (Grimm and Ram, 2009; Frankfurt et al., 2016). Latent classes are unobserved groups that emerge from the data depending on the patterns of growth in GMM. Subgroups with their own unique growth parameters are identified as illustrated in Figure 1C (latent classes *c* represented by a circle as an unobserved categorical variable and their specific means of intercept and slope factors denoted by paths f and g). For example, Cabrera et al. (2016) identified four distinctive trajectories of post-combat aggression among American combat team soldiers returned from an Iraq deployment: low-stable, delayed, recovery, and chronic. They expected that their study findings could help targeted intervention of combat-related posttraumatic stress disorder through improved identification of at-risk subgroups. In addition to identifying subpopulations of heterogeneous growth curves, GMM is used to approximate non-normal distributions (McLachlan and Peel, 2000; Lubke and Neale, 2006). Normal distribution is typically assumed within subpopulation (Muthén, 2004) and the distribution of observed variables is the mixture distribution of subpopulations (Lubke and Neale, 2006). As in LGM, researchers can incorporate covariates in GMM although not demonstrated in the figure. For interested readers, refer to Muthén (2004) about the extension of GMM with covariates and distal outcomes.

When subpopulations are identified in GMM, it is assumed that measurement invariance of longitudinal outcome variables holds across identified subpopulations. For example, soldiers showing low-stable post-combat aggression and soldiers showing chronic post-combat aggression (Cabrera et al., 2016) are assumed to respond to the items of the aggression scale in the same way. However, this assumption can be violated as illustrated in Figure 1D. The figure shows that there is heterogeneity across latent classes not only in baseline performance and growth rates (paths h and i, respectively) but also in their responses to an item (path j). As discussed in the LGM section above, it is well-known that scalar invariance between groups is a prerequisite to a meaningful group mean comparison. Similarly, a comparison between latent classes in terms of means of intercept and slope factors (or initial performance and growth) is expected to be meaningfully interpretable when measurement invariance holds. Based on the findings of Kim and Willson (2014a) with LGM, when measurement noninvariance across latent classes is present but invariance is assumed in GMM, it is possible that spurious heterogeneity in growth parameters occurs, which can result in the detection of a spurious latent class. It is also reported that assumption violations could lead to the misidentification of an extra latent class (Bauer and Curran, 2003). Particularly, specifying a more restrictive model than a true population model within class could result in overestimation of the number of classes (Lubke and Neale, 2008; Vermunt, 2011). For example, four latent classes may be identified when there are three distinctive classes in the population. However, the impact of measurement noninvariance across latent classes on the performance of GMM has not been systematically studied yet.

### Second-order growth mixture modeling

In GMM, longitudinal outcome variables are observed variables. Many applied studies using GMM employed mean or sum composite scores of multiple items of a scale (e.g., mean of eight items of 3-point peer victimization scale, Brendgen et al., 2016; sum of five items of 4-point positive religious coping scale and sum of five items of 4-point negative religious coping scale, Hayward and Krause, 2016; sum of five items of 5-point self-esteem scale, Oshri et al., 2017; mean of 16 items of 5-point depressive symptoms, Wang et al., 2015). On the other hand, the second-order GMM (SOGMM) directly models the relation of multiple items to the factor that is repeatedly measured for changes as illustrated in Figure 1D. Grimm and Ram (2009) described SOGMM by decomposing the model into four components: (1) a longitudinal measurement model or longitudinal common factor model, (2) measurement invariance constraints, (3) a latent growth model, and (4) a mixture model. On top of GMM (components 3 and 4) which is the second-order part, SOGMM includes a measurement model at each time point as the first-order part (components 1 and 2). Thus, the benefits of SOLGM over LGM we listed above will equally apply to SOGMM over GMM and we do not reiterate those benefits here. Of note is that Grimm and Ram demonstrated the application of SOGMM with multiple assessment (different reporters of a measure, that is, mother, father, and teacher reports of child externalizing behavior) not with multiple items of a measure. They still used sum composite scores of multiple items of mother, father, and teacher reports, and a measurement model of each scale was not employed in their SOGMM. Following their demonstration, some applications of SOGMM included multiple assessment in the measurement model with composite scores not with multiple items of a measure (e.g., Nash et al., 2015; Lee et al., 2017). Of another note is measurement invariance constraints. When measurement invariance holds over time, invariance constraints are imposed as illustrated in Grimm and Ram and also in Figure 1D (denoted by k, l, and m over occasions). In their demonstration measurement invariance *across latent classes* were assumed (Grimm and Ram, 2009). That is, a path from latent classes to an item (or multiple assessment) denoted by path j was constrained at zero for all items. However, in SOGMM this path, that is, measurement invariance of the corresponding item can be explicitly tested and also freely estimated when the invariance assumption does not hold. Even though SOGMM has great flexibility in testing and modeling heterogeneity across latent classes not only in growth patterns but also in measurement models, its application is not common and very limited (only with multiple assessment not with items) up to date. Moreover, the efficacy of SOGMM in detecting heterogeneous subpopulations is unknown.

Given that research on the performance of GMM and second-order GMM in the presence of measurement noninvariance is lacking, the purpose of this study is two-fold. In Study 1, we purport to investigate the impact of measurement noninvariance on class enumeration and parameter recovery in GMM. Specifically, when population heterogeneity exists in terms of measurement parameters but the scale composite scores are used in GMM ignoring measurement noninvariance, how the violation of measurement invariance affects the class enumeration and parameter estimates is examined through a Monte Carlo simulation study. In Study 2, we examine the class enumeration accuracy and parameter recovery of SOGMM in which measurement parameters are allowed to vary across latent classes.

## Theoretical framework

### Latent growth modeling

With data from repeated measures researchers can investigate growth trajectories such as the average performance at the initial stage and the average growth rate across individuals. The common factor model in structural equation modeling (SEM) can be used to address such research interest. In the SEM framework, growth trajectories such as baseline performance and growth are modeled as latent variables. As shown in Figure 1A, baseline performance and growth are represented by the intercept and slope latent factors, respectively in LGM. For the sake of simplicity, we assume linear growth in this example, but the model can be easily extended to different growth curves by including additional latent factors (e.g., a quadratic factor for curvilinear growth) or freely estimating factor loadings of the slope factor. The intercept and slope (**ξ**_*i*_) are estimated with observed continuous outcome variables of repeated measures (*T*_*i*_) for an individual *i* (Meredith and Tisak, 1990; Wu et al., 2009) as shown in Equation (1).


(1) $$T_{i}=\Gamma \xi_{i}+\zeta_{i}$$

where *T*_*i*_ is a *m* × 1 vector of observed variables, Γ is a *m* × *r* matrix of factor loadings, **ξ**_*i*_ is a vector of latent factor scores (i.e., intercept and slope values), **ζ**_*i*_ is a *m* × 1 vector of time-specific error scores for an individual *i, m* is the number of occasions, and *r* is the number of latent factors (2 with the intercept and slope factors). For linear growth over time, the factor loadings of the intercept and slope factors can be specified as:


$$\Gamma =\left[\begin{array}{c} \begin{array}{c} 1\\ 1 \end{array}\\ \vdots \\ 1 \end{array}\begin{array}{c} \begin{array}{c} 0\\ 1 \end{array}\\ \vdots \\ \;m-1 \end{array}\right].$$

The factor loadings of the intercept factor are all unity and those of the slope factor increase by unity from 0 to *m* − 1 to represent linear growth over *m* occasions. The subscript *i* in the intercept and slope factors (**ξ**_*i*_) indicates that individuals are allowed to have different intercepts (initial performance) and slopes (linear growth rates), but the average is of focal interest. The means of two latent factors, *E*(**ξ**_*i*_) are expressed as:


(2) $$E(\xi_{i})\;=\kappa =\left[\begin{array}{c} \kappa_{I}\\ \kappa_{S} \end{array}\right]$$

where κ_*I*_ and κ_*S*_ represent the average baseline performance and the average growth rate across individuals, respectively. The variance covariance matrix of the latent factors is:


$$\Phi =\left[\begin{array}{cc} \phi_{I} & \\ \phi_{IS} & \phi_{S} \end{array}\right]$$

where ϕ_*I*_, ϕ_*S*_, and ϕ_*IS*_ represent the variability in baseline performance and growth across individuals and the covariance between baseline performance and growth, respectively. Finally, the population mean vector μ_*T*_ and variance covariance matrix Σ_*T*_ of *T*_*i*_ are defined as:


(3) $$\begin{array}{l} \mu_{T}=\Gamma \kappa \\ \Sigma_{T}=\Gamma \Phi \Gamma^{\prime }+\Psi \end{array}$$

where Ψ is the variance covariance matrix of residuals (**ζ**_*i*_). The residuals (**ζ**_*i*_) are assumed to be multivariate normally distributed with the mean of zero and independent of each other, but the assumption of independence can be relaxed by allowing residual covariance. Of note is that in LGM applications the observed outcome variables (***T***_*i*_,) are typically mean or sum composite scores of a measure.

### Growth mixture modeling

To model the differences specifically in growth trajectories across individuals, GMM incorporates latent classes in LGM. Thus, GMM includes both latent continuous variables (latent factors) and latent categorical variables (latent classes; Muthén, 2004). The latent growth model introduced in Equation (1) will be specified for each latent class as shown below.


(4) $$(T_{i}|c)=\Gamma_{c}\xi_{ic}+\zeta_{ic}$$

where *c* denotes latent classes (*c* = 1, 2, …, *C*). Within class, the residuals (ζ_*i*_|*c*) are assumed to be multivariate normally distributed with a mean vector of 0 and variance covariance matrix of Ψ_*c*_. Accordingly, Equations (2) and (3) are rewritten as


$$\begin{array}{l} \kappa_{c}=\left[\begin{array}{c} \kappa_{Ic}\\ \kappa_{Sc} \end{array}\right],\\ \mu_{Tc}=\Gamma_{c}\kappa_{c},\\ \Sigma_{Tc}=\Gamma_{c}\Phi_{c}{\Gamma_{c}}^{\prime }+\Psi_{c}. \end{array}$$

Thus, all parameters of LGM such as the intercept and slope factor means (κ_*I*_ and κ_*S*_) and their variances and covariance (ϕ_*I*_, ϕ_*S*_, and ϕ_*IS*_) can be class specific in GMM. In addition, the probability that an individual belongs to each category of latent classes is estimated. Hence, the distribution of the longitudinal outcome variables is a mixture of normal distributions of latent classes as shown below:


(5) $$f(T_{i})=\sum_{c=1}^{C}\pi_{c}\varphi_{c}(\mu_{c},\;\Sigma_{Tc})$$

where φ_**c**_ is a *m*-dimensional normal probability density function for class *c*, π_*c*_ is the proportion of participants in class *c*, and $\overset{C}{\underset{c=1}{\sum }}\pi_{c}=1$ (Bauer, 2007).

### Measurement invariance testing in the second-order growth mixture model

With repeated measures, the second-order growth mixture model (SOGMM) that incorporates a measurement model at the first-order level has advantages over GMM that usually uses composite scores of repeated measures. SOGMM takes into account measurement error (residuals of items not related to a common factor) and allows researchers to evaluate psychometric qualities of a scale including measurement invariance across latent classes. Thus, SOGMM is appropriate to detecting unknown clustering due to noninvariance in measurement parameters of a scale as well as heterogeneity in growth trajectories among individuals.

As illustrated in Figure 1D, the first-order part of SOGMM is a measurement model at each occasion *t* that models the relation of observed continuous variables to latent factors (e.g., depressive symptoms items to a latent factor depressive symptoms):


(6) $$(Y_{it}|c)=\nu_{tc}+\Lambda_{tc}\eta_{itc}+\varepsilon_{itc}$$

where conditional on latent class *c*, *Y*_*it*_ is a *p* × 1 vector of continuous observed variables (or items), **ν**_*tc*_ is a *p* × 1 vector of item intercepts, **Λ**_*tc*_ is a *p* × *q* matrix of item factor loadings, **η**_*itc*_ is a *q* × 1 vector of latent factor scores, **ε**_*itc*_ is a *p* × 1 vector of the corresponding item error scores for an individual *i*, and *p* and *q* are the number of items and the number of factors, respectively. Within class, the residuals are assumed to be multivariate normally distributed with a mean vector of 0: (**ε**_*it*_|*c*) ~ *N*(0, **Θ**_*tc*_).

Because measurement models are explicit in SOGMM, measurement invariance over time can be specified. Strict measurement invariance holds over time for class *c* if


$$\Lambda_{tc}=\Lambda_{c},\;\nu_{tc}=\nu_{c},\;\Theta_{tc}=\Theta_{c}.$$

Similarly, measurement invariance across latent classes can be specified in SOGMM. If measurement invariance over time holds as shown above, strict measurement invariance across latent classes (*c* = 1, 2, …, *C*) can be further defined as:


(7) $$\begin{array}{l} \Lambda_{c}=\Lambda , \end{array}$$

(8) $$\begin{array}{l} \nu_{c}=\nu , \end{array}$$

(9) $$\begin{array}{l} \Theta_{c}=\Theta . \end{array}$$

When strict invariance holds across classes, factor variances and means are freely estimated and compared across classes (or over time).

Then, the second-order part of SOGMM is basically GMM that is shown in Equation (4). To thread the first- and second-order parts of SOGMM together, the measurement model at occasion *t* in Equation (6) is rewritten as a measurement model over *t* occasions without the *t* subscript: (**Y**_*i*_|*c*) = **ν**_*c*_ + **Λ**_*c*_**η**_*ic*_ + **ε**_*ic*_. By replacing η_*ic*_ with (*T*_*i*_|*c*) in Equation (4), the measurement model and the growth mixture model (Equation 4) are combined as the second-order growth mixture model (Figure 1D):


$$\left(Y_{i}|c\right)=\nu_{c}+\Lambda_{c}\left(\Gamma_{c}\xi_{ic}+\zeta_{ic}\right)+\varepsilon_{ic}.$$

The mean vector μ_*Yc*_ and variance covariance matrix **Σ**_*Yc*_ of (***Y***_*i*_|*c*) are defined as


(10) $$\begin{array}{l} \mu_{Yc}=\nu_{c}+\Lambda_{c}\Gamma_{c}\kappa_{c},\\ \Sigma_{Yc}=\Lambda_{c}\left(\Gamma_{c}\Phi_{c}\Gamma_{c}^{\prime }+\Psi_{c}\right)\Lambda_{c}^{\prime }+\Theta_{c}. \end{array}$$

When GMM is used with composite scores of repeated measures, measurement noninvariance, if present, cannot be properly modeled. Kim and Willson (2014a) showed that when measurement noninvariance was present between groups but not correctly modeled by constructing latent growth models with composite scores, ignoring measurement noninvariance resulted in biased estimates of baseline performance and growth factor means and incorrect statistical inferences on these parameters. Specifically, they found that noninvariance in factor loadings led to a spurious mean difference in growth between groups whereas noninvariance in intercepts yielded a spurious mean difference in baseline performance. The size of measurement noninvariance ignored in LGM was directly related to the size of bias in those mean differences. In Appendix (Supplementary Material) we analytically demonstrated the impact of ignored measurement noninvariance on the estimates of growth factor means using SOGMM.

### Class enumeration in growth mixture modeling

In practice of mixture modeling, a series of models with an increasing number of latent classes are specified. Then, the number of latent classes is commonly determined by identifying the best-fitting model among all specified models through model comparisons. To select the best-fitting model, different methods are introduced in the literature. Tein et al. (2013) summarized class enumeration methods into three categories: (a) using information criterion (IC) such as the Akaike Information Criterion (AIC; Akaike, 1974), Consistent AIC (CAIC; Bozdogan, 1987), Bayesian Information Criterion (BIC; Schwarz, 1978), and sample-size adjusted BIC (saBIC; Sclove, 1987), (b) conducting likelihood ratio tests (LRT) such as Lo-Mendell-Rubin LRT and bootstrap LRT, and (c) using entropy that evaluates how well the classes are separated. In this study we use information criteria for class enumeration. Among ICs, Nylund et al. (2007) recommended BIC and saBIC for class enumeration in GMM. These two ICs are also commonly used and suggested in the general mixture modeling literature (e.g., Lubke and Muthén, 2005; Tay et al., 2011). However, some authors showed the outperformance of AIC over BIC particularly when sample size was small and the class separation was poor (Lukočienė et al., 2010), but the AIC tended to overestimate the number of latent classes in other cases (Celeux and Soromenho, 1996; Nylund et al., 2007; Tein et al., 2013). In model selection with mixture modeling, the hierarchical BIC (HBIC) is also suggested (Zhao et al., 2013, 2015; Gollini and Murphy, 2014; Zhao, 2014). Zhao et al. (2015) argued that BIC tends to overpenalize model complexity in mixture modeling by using the total sample size for all estimated parameters and suggested to penalize parameters with their relevant sample size, that is, local or effective sample size that is used to estimate parameters associated with a specific class (*nπ*_*c*_ in the equation below). As shown in Equation (11), the HBIC equals to the BIC when *c* = 1, but is smaller than BIC when *c* > 1. Zhao and colleagues demonstrated that the HBIC outperformed the BIC especially when sample size was small. Thus, we included these four ICs in our study. These ICs are computed as:


(11) $$\begin{array}{l} \text{ AIC}=-2\text{log}L+2^{*}k,\\ \text{ BIC}=-2\text{log}L+log{\left(n\right)}^{*}k,\\ \text{saBIC}=-2\text{log}L+log{\left[(n+2)/24\right]}^{*}k,\\ \text{HBIC}=-2\text{log}L+\;\left(k_{0}+C-1\right)\log\left(n\right)+\sum_{c=1}^{C}\log\left(n\pi_{c}\right)*k_{c}^{\prime } \end{array}$$

where log*L* means log likelihood, log(*n*) is the natural logarithm of sample size, log(*nπ*_*c*_) is the natural logarithm of sample size specific to a latent class *c* where π_*c*_ ≥ 0, *c* = 1, 2, …, *C*, and $\overset{C}{\underset{c=1}{\sum }}\pi_{c}=1$, *k* and $k_{c}^{\prime }$ represent the number of freely estimated parameters for the total sample and for a latent class *c*, respectively, and *k*_0_ is the number of free parameters common across latent classes (hence, $k=k_{0}+C-1+\overset{C}{\underset{c=1}{\sum }}k_{c}^{\prime }$).

This study investigated how measurement noninvariance in a scale across latent classes makes impact on class enumeration and parameter estimates when GMM is used to evaluate growth over time ignoring the lack of invariance. In addition, when SOGMM is used, that is, measurement models are incorporated in GMM and measurement parameters are allowed to be heterogeneous across latent classes, the class enumeration accuracy and bias of parameter estimates in SOGMM was examined in the presence of measurement noninvariance. We hypothesize the following:

1. When baseline performance and growth are homogeneous on average, that is, latent classes are not present in terms of the intercept and slope factor means, GMM would falsely identify latent classes because the ignored measurement noninvariance would be detected as heterogeneity in these factor means.
2. When latent classes are falsely identified, a spurious mean difference in the slope factor would be observed if there is noninvariance in factor loadings; a spurious mean difference in the intercept factor would be observed if there is noninvariance in intercepts. The size of the spurious mean difference would be associated with the size of ignored measurement noninvariance.
3. SOGMM would correctly identify the number of latent classes in the presence of measurement noninvariance.
4. SOGMM would yield unbiased estimates of the difference between latent classes with respect to the intercept and slope factor means in the growth model part as well as factor loadings and intercepts in the measurement model part.

## Study 1: growth mixture modeling in the presence of measurement noninvariance between latent classes

### Method

We conducted a Monte Carlo simulation study to investigate the impact of measurement noninvariance on the class enumeration and parameter recovery of GMM. The simulation factors included (a) location of noninvariance (factor loading/intercept), (b) degree of noninvariance (small/large), (c) difference in the intercept and slope factor means (zero/large), (d) sample size (100/200/400/1000), and (e) mixing proportion (balanced/unbalanced). Because the impact of measurement noninvariance on the performance of GMM was of focal interest in this study, the following factors were fixed as a constant for simplicity of discussions: two latent classes, four occasions, six items that load on a single factor at each occasion, and two noninvariant items, which were commonly adopted in previous simulation studies (e.g., Nylund et al., 2007; Chen et al., 2010; Kim and Willson, 2014a). In addition, measurement invariance over time was simulated. Although temporal invariance can also be violated in reality, the impact of noninvariance across latent classes could be less clear to delineate when noninvariance is present at both locations. Of note is that measurement invariance over time can be tested separately with a longitudinal common factor model and, if invariance holds, researchers can impose temporal invariance constraints on SOGMM which was demonstrated by Grimm and Ram (2009). However, measurement invariance across classes cannot be tested separately because latent classes are unobservable in advance. Of another note is that we investigated the impact of measurement noninvariance in factor loadings and intercepts between latent classes (violation of Equations 7 and 8) because scalar invariance is considered as a prerequisite to meaningful mean comparisons across groups (Millsap and Kwok, 2004; Raykov et al., 2012; Jak et al., 2014). Finally, error correlations over time were not simulated for the simplicity because this is not a major interest in this study.

#### Data generation

**Figure 2:**
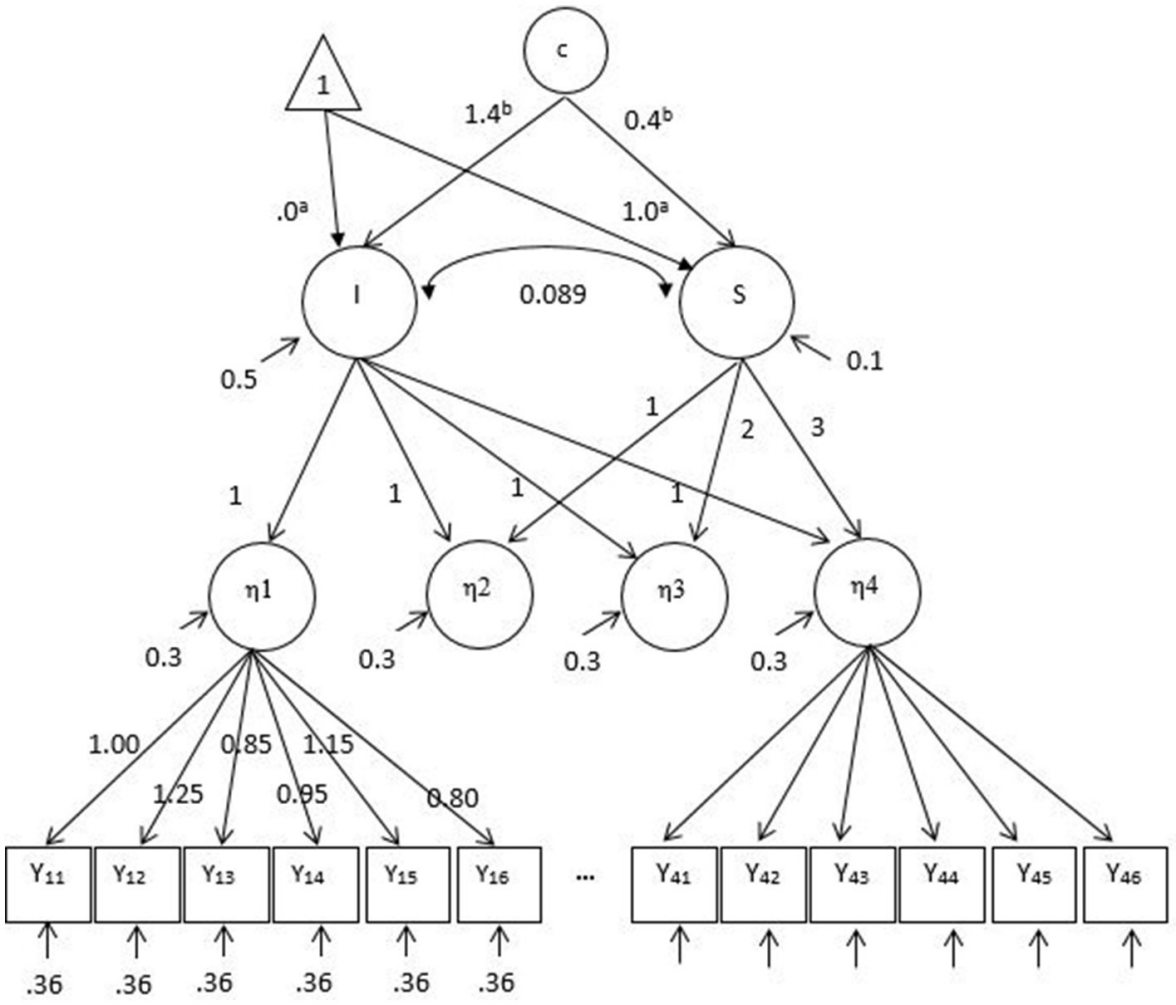
Population parameters for data generation with the second-order growth mixture model under the factor mean difference conditions. A linear growth over time is generated. I, Latent intercept; S, latent slope; c, unobserved categorical variable or latent classes. For simplicity the measurement intercept values are not specified in this figure. Y_11_–Y_46_ are observed items of latent factors, η1–η4. Note that Y_21_–Y_36_ are not shown due to a limited space. The same set of factor loadings and residual variances of six items are applied over time for η1–η4. ^a^The intercept and slope factor means of a latent class (i.e., reference class), respectively. ^b^The mean differences between latent classes for the intercept and slope factors, respectively when the number of classes is two.

Data were generated using the second-order growth mixture model with a measurement model at each occasion of repeated measures. The population parameters used for data generation are presented in Figure 2. The parameters in the first-order measurement model were majorly adopted from Kim and Willson (2014a) who conducted a similar study with observed groups using the second-order LGM. The generated values of factor loadings (0.80 ~ 1.25), intercepts (−0.15 ~ 0.25), and residual variances (0.36) were also observed in previous simulation studies of measurement invariance (Wirth, 2009; Kim et al., 2012). In the second-order latent growth model, a linear growth over four occasions was simulated. The means of baseline performance and growth factors (or intercept and slope factors) were 0 and 1, respectively. The respective variances were 0.5 and 0.1, and their covariance was 0.089 which corresponded to correlation 0.4 (Leite, 2007). The ratio of the intercept factor variance to the slope factor variance, 5:1 is considered reasonable in practice and adopted in other simulation studies (Muthén and Muthén, 2002; Depaoli, 2013; Li and Harring, 2016). The reliability estimates of 24 generated items (6 items per occasion) ranged from 0.59 to 0.91.

In the first-order measurement model, two out of six items were simulated as noninvariant across all simulation conditions. The 0.20 and 0.40 differences between classes for small and large factor loading noninvariance, respectively, and the 0.30 and 0.60 differences for small and large intercept noninvariance, respectively, were generated (e.g., Stark et al., 2006). On top of measurement noninvariance, population heterogeneity was simulated in the mean of intercept and slope factors at two levels (zero or large). When there was no difference between two classes in the intercept and slope factor means, both classes had the intercept and slope factor means of 0 and 1, respectively. When a large difference between latent classes was generated, the intercept and slope factor means of the second class were higher by 1.4 and 0.4, respectively and thus this class performs better at the baseline and also grows faster over time. The generated mean differences corresponded to Mahalanobis distance (MD) 2.0. In the mixture literature, MD 2.0 is considered as large class separation (e.g., Tueller and Lubke, 2010; Depaoli, 2013; Li and Harring, 2016). Of note is that class separation is one of major factors associated with the correct enumeration of latent classes (Henson et al., 2007; Tofighi and Enders, 2008; Chen et al., 2010).

The combination of two simulation factors, that is, (a) noninvariance in measurement parameters and (b) mean differences in the intercept and slope factors yielded two types of population heterogeneity. When there was no factor mean difference, measurement noninvariance was the only source of population heterogeneity that differentiated two latent classes. When there were factor mean differences, two sources of population heterogeneity, that is, measurement noninvariance and factor mean differences separated two latent classes. In the latter, the latent class with higher item factor loadings had higher factor means under the factor loading noninvariance conditions; the latent class with higher item intercepts had higher factor means under the intercept noninvariance conditions. By generating data in this way (positive pairing), we expected that the factor mean differences between latent classes would be overestimated when the invariance was assumed because the ignored noninvariance could make the factor mean of the higher class even higher (the factor mean of the lower class even lower). This was illustrated in Appendix (Supplementary Material). When two latent classes were disproportionately formed, the mixing proportion was 80 and 20%. The latent class with higher factor means and/or measurement parameters was associated with a large sample size (i.e., 80%) when two latent classes were unbalanced. For each condition, 500 replications were generated using Mplus version 7.3 (Muthén and Muthén, 2014).

#### Fitted models and simulation outcomes

In Study 1, the fitted model was a growth mixture model in which measurement noninvariance could not be modeled although the data were generated with measurement noninvariance. The mean composite scores were used as observed indicators of the growth mixture model ignoring noninvariance of items. It should be noted that equal weights were applied for all items when composite scores were created although factor loadings were different across items in the population. A linear growth was modeled with factor loadings of the intercept factor all fixed at 1 and those of the slope factor specified at 0, 1, 2, and 3 for four occasions. Because there were two latent classes in the population, models with one, two, and three latent classes were evaluated and a best fitting model was selected on the basis of the selected fit index (i.e., AIC, BIC, saBIC, and HBIC). Note that a latent class with a cell proportion < 0.05 was ruled out because the number of observations in a class (e.g., less than five observations with *N* = 100) was too small (e.g., Feldman et al., 2009). For example, even though the ICs supported three classes, if one of them constituted <5% of total observations, this replication was counted as two classes. The class enumeration was recorded for each replication and the enumeration rates for one-, two-, and three-class models were computed by simulation conditions and fit indexes. The enumeration rate of two classes, for example, was computed by dividing the number of replications that supported a two-class model by the total number of replications.

When no factor mean difference was simulated in the growth model (precisely speaking, the second-order part of SOGMM), one class was considered as a correct number of classes. However, we hypothesized that two classes would emerge due to ignored measurement noninvariance between two classes as observed in Kim and Willson (2014a; that is, a factor mean difference was detected when there was no factor mean difference between two groups). When two classes were generated with different factor means in the intercept and slope factors, two classes were expected to be detected correctly. However, in either scenario (one class or two classes), we hypothesized that the parameter estimates of GMM would be biased due to ignored measurement noninvariance in the measurement model. Specifically, we examined the bias in the means of the intercept and slope factors. The bias was estimated as the average difference between the estimated factor mean and the generated population factor mean across replications. If the population parameter was not zero, we also estimated relative bias which is the ratio of the estimated bias to the population parameter. Relative bias >0.05 is typically considered substantial in the simulation studies (Hoogland and Boomsma, 1998). Standardized bias was not considered because it is possibly affected by sample size (the larger sample size, the smaller standardized bias holding raw bias constant). Although, the values of raw bias are less interpretable, raw bias would suffice to show the impact of measurement noninvariance on the estimates of GMM. GMM was fitted with Mplus version 7.3 (Muthén and Muthén, 2014).

### Results

#### Class enumeration

The class enumeration rates of AIC, BIC, saBIC, and HBIC for the balanced conditions are presented in Table 1. The enumeration rates for the unbalanced conditions are similar and, thus, not presented here. The top panel of Table 1 showed the enumeration rates of GMM when there was no heterogeneity in the intercept and slope factor means. Thus, one class was considered as a correct number of classes. However, because GMM used mean composite scores ignoring measurement noninvariance between two latent classes, we hypothesized that two classes would emerge. Unexpectedly, one class was generally selected, which might indicate that GMM was not very sensitive to the ignored measurement noninvariance. The BIC and HBIC identified one class as a best fit model almost always regardless of simulation factors. The saBIC also selected one class, but less frequently as sample size decreased (e.g., 0.97 with *N* = 1,000 and 0.28 with *N* = 100 when small noninvariance was simulated in factor loadings). The AIC was not affected by simulation factors much: the enumeration rates for a one-class model were around 0.60 across simulation conditions.

**Table 1:** The class enumeration rates of growth mixture modeling for the balanced conditions.

**DIF location**	**DIF size**	**Sample size**	**AIC**			**BIC**		**saBIC**			**HBIC**	
			**Number of latent classes**									
			**1**	**2**	**3**	**1**	**2^a^**	**1**	**2**	**3**	**1**	**2^a^**
**NO FACTOR MEAN DIFFERENCE**												
Loading	Small	50/50	**0.56**	0.28	0.16	**0.99**	0.01	**0.28**	0.41	0.30	**0.89**	0.09^a^
		100/100	**0.65**	0.23	0.11	**0.99**	0.01	**0.71**	0.19	0.10	**0.97**	0.02^a^
		200/200	**0.68**	0.24	0.08	**1.00**	0.00	**0.91**	0.08	0.02	**0.99**	0.01
		500/500	**0.72**	0.23	0.05	**1.00**	–	**0.97**	0.03	0.00	**0.99**	0.01
	Large	50/50	**0.55**	0.29	0.16	**0.99**	0.01	**0.28**	0.42	0.31	**0.90**	0.08^a^
		100/100	**0.64**	0.27	0.09	**1.00**	0.00	**0.69**	0.23	0.08	**0.96**	0.03^a^
		200/200	**0.67**	0.25	0.08	**1.00**	–	**0.89**	0.09	0.02	**0.99**	0.01
		500/500	**0.68**	0.27	0.05	**1.00**	0.00	**0.95**	0.05	0.00	**0.99**	0.01
Intercept	Small	50/50	**0.57**	0.26	0.17	**0.99**	0.01	**0.30**	0.39	0.31	**0.89**	0.09^a^
		100/100	**0.64**	0.25	0.11	**0.99**	0.01	**0.69**	0.23	0.08	**0.97**	0.02^a^
		200/200	**0.69**	0.25	0.06	**1.00**	0.00	**0.90**	0.08	0.02	**0.99**	0.01
		500/500	**0.74**	0.22	0.04	**1.00**	–	**0.97**	0.02	0.00	**1.00**	0.00
	Large	50/50	**0.59**	0.24	0.17	**0.99**	0.01	**0.31**	0.37	0.32	**0.90**	0.09^a^
		100/100	**0.63**	0.26	0.11	**0.99**	0.01	**0.68**	0.23	0.09	**0.97**	0.03^a^
		200/200	**0.68**	0.26	0.06	**1.00**	0.00	**0.90**	0.08	0.02	**0.99**	0.01
		500/500	**0.75**	0.20	0.05	**1.00**	–	**0.97**	0.03	–	**1.00**	0.00
**FACTOR MEAN DIFFERENCE**												
Loading	Small	50/50	0.36	**0.40**	0.24	0.94	**0.06** ^a^	0.17	**0.44**	0.39	0.80	**0.16** ^a^
		100/100	0.19	**0.63**	0.18	0.91	**0.09**	0.22	**0.63**	0.16	0.80	**0.18** ^a^
		200/200	0.08	**0.75**	0.17	0.78	**0.22**	0.21	**0.72**	0.07	0.63	**0.37** ^a^
		500/500	0.00	**0.80**	0.20	0.21	**0.79**	0.02	**0.94**	0.04	0.12	**0.87** ^a^
	Large	50/50	0.25	**0.48**	0.27	0.87	**0.12** ^a^	0.09	**0.48**	0.43	0.70	**0.24** ^a^
		100/100	0.10	**0.65**	0.25	0.79	**0.21** ^a^	0.13	**0.65**	0.22	0.60	**0.37** ^a^
		200/200	0.01	**0.67**	0.32	0.42	**0.57** ^a^	0.03	**0.79**	0.18	0.29	**0.68** ^a^
		500/500	–	**0.52**	0.48	0.00	**0.98** ^a^	–	**0.78**	0.22	0.00	**0.90** ^a^
Intercept	Small	50/50	0.40	**0.40**	0.20	0.94	**0.06**	0.20	**0.45**	0.35	0.82	**0.16** ^a^
		100/100	0.31	**0.55**	0.13	0.93	**0.07**	0.35	**0.53**	0.11	0.84	**0.16**
		200/200	0.13	**0.71**	0.17	0.83	**0.17**	0.28	**0.67**	0.05	0.73	**0.27**
		500/500	0.00	**0.90**	0.09	0.35	**0.65**	0.04	**0.94**	0.02	0.23	**0.77**
	Large	50/50	0.32	**0.46**	0.22	0.92	**0.08** ^a^	0.12	**0.49**	0.39	0.78	**0.20** ^a^
		100/100	0.23	**0.58**	0.19	0.88	**0.12**	0.27	**0.57**	0.15	0.78	**0.22** ^a^
		200/200	0.06	**0.80**	0.15	0.74	**0.26**	0.17	**0.78**	0.05	0.61	**0.39** ^a^
		500/500	–	**0.87**	0.13	0.18	**0.82**	0.01	**0.96**	0.03	0.10	**0.90** ^a^

*The hypothesized correct enumeration rates are in bold. DIF, Differential item functioning or measurement noninvariance; AIC, Akaike information criterion; BIC, Bayesian information criterion; saBIC, sample-size adjusted BIC; HBIC, hierarchical BIC. Due to rounding 0.00 means one or two replications out of 500*.

a *We compared one-, two-, and three-class models, and the three-class model was selected with a small proportion*.

The bottom panel of Table 1 showed the enumeration rates of GMM when differences in the intercept and slope factor means were simulated between two classes in addition to measurement noninvariance. The model with two latent classes was expected to be selected. However, the BIC and HBIC still selected one class more frequently showing its insensitivity to population heterogeneity when sample size was not large. Only when sample size reached 1,000 and the size of noninvariance was large, two classes were mostly identified. We could observe the impact of the unmodeled measurement noninvariance on the enumeration rates of BIC and HBIC. Specifically, the enumeration accuracy of BIC and HBIC depended on the size and location of noninvariance. When factor loadings were noninvariant, both selected two classes more frequently compared to the intercept noninvariance conditions. As the size of noninvariance increased, the correct enumeration rates also increased. This possibly implies that the ignored measurement noninvariance created larger class separation by adding its unmodeled effect to the factor mean differences in GMM. This impact of measurement noninvariance was not observed with the AIC and saBIC. In general, the correct enumeration rates of the saBIC were higher than those of the AIC, BIC, and HBIC. The HBIC outperformed the BIC, but it sometimes over-identified latent classes (i.e., three classes). For all four information criteria, it was prominent that the enumeration accuracy was associated with sample size (the larger, the more accurate). We also examined the class proportion when two classes were selected. The class proportion was generally consistent to the population proportion (that is, 50 and 50% for the balanced conditions and 20% and 80% for the unbalanced conditions).

#### Bias and relative bias

Bias and relative bias were examined with the replications in which the number of classes was correctly identified (see Table 2). It should be noted that the raw bias of the intercept and slope factor means is presented for no factor mean difference conditions because the intercept factor mean is zero (i.e., relative bias cannot be computed). For factor mean difference conditions the relative bias of the differences is presented (the intercept factor mean difference = 1.4; the slope factor mean difference = 0.4). Across conditions, three patterns emerged. First, only the factor means associated with the ignored noninvariance were biased whereas the factor means not associated with the ignored noninvariance were unbiased with raw and relative bias close to zero. Specifically, when noninvariance was simulated in the factor loadings of the first-order measurement model but ignored in GMM, the slope factor means showed notable bias while the intercept factor means remained unbiased; when noninvariance was simulated in the intercepts of the first-order measurement model but ignored in GMM, the intercept factor means were biased while the slope factor means were unbiased. Second, the magnitude of bias was directly related to the magnitude of ignored measurement noninvariance irrespective of sample size. This pattern was clearly observed in the raw bias under no factor mean difference conditions. When the magnitude of noninvariance was doubled, the magnitude of bias in factor means was also doubled. For example, for the balanced conditions with ignored intercept noninvariance, the raw bias in the intercept factor means was about 0.05 when small noninvariance was ignored and about 0.10 when large noninvariance was ignored. Third, the direction of bias also reflected the direction of ignored measurement noninvariance. Under the no factor mean difference conditions, two factor loadings were simulated lower in one class, which probably led to negative bias in the slope factor means whereas two intercepts were simulated higher in one class, which conceivably resulted in positive bias in the intercept factor means. Under the factor mean difference conditions, noninvariance, regardless of its location, was simulated in favor of the class with higher factor means (positive pairing; the latent class with higher item factor loadings had higher factor means under the factor loading noninvariance conditions; the latent class with higher item intercepts had higher factor means under the intercept noninvariance conditions). As hypothesized and illustrated in Appendix (Supplementary Material), the corresponding factor mean differences were mostly positively biased because the factor mean of the class with a higher factor mean tended to be overestimated and that of the class with a lower factor mean tended to be underestimated. Compared to the balanced conditions, the parameter estimates in the unbalanced conditions were more biased when intercepts were not invariant, but less biased when factor loadings were not invariant.

**Table 2:** The bias and relative bias of the intercept and slope factor means in growth mixture modeling.

**DIF location**	**DIF size**	**Sample size**	**No difference (Raw bias)**		**Difference (Relative bias)**	
			**Intercept**	**Slope**	**Intercept *d***	**Slope *d***
Loading	Small	50/50	0.006	−0.034	−0.003	−0.210
		100/100	0.004	−0.034	0.002	0.035
		200/200	0.005	−0.033	−0.003	0.125
		500/500	0.002	−0.033	0.009	0.165
	Large	50/50	0.006	−0.067	0.001	0.182
		100/100	0.004	−0.067	0.010	0.317
		200/200	0.004	−0.066	0.019	0.345
		500/500	0.002	−0.066	0.017	0.360
Intercept	Small	50/50	0.057	0.000	0.054	−0.510
		100/100	0.055	−0.001	0.083	−0.155
		200/200	0.055	0.001	0.089	−0.053
		500/500	0.051	0.000	0.071	0.001
	Large	50/50	0.107	0.000	0.110	−0.108
		100/100	0.105	−0.001	0.165	−0.028
		200/200	0.105	0.001	0.151	−0.020
		500/500	0.101	0.000	0.145	−0.008
Loading	Small	80/20	0.006	−0.014	0.007	−0.245
		160/40	0.004	−0.014	0.017	0.006
		320/80	0.005	−0.013	−0.009	0.114
		800/200	0.002	−0.013	−0.035	0.113
	Large	80/20	0.006	−0.027	−0.058	−0.080
		160/40	0.004	−0.027	−0.015	0.138
		320/80	0.004	−0.026	−0.044	0.237
		800/200	0.002	−0.026	−0.065	0.238
Intercept	Small	80/20	0.087	−0.001	0.081	−0.270
		160/40	0.085	−0.001	0.118	−0.043
		320/80	0.085	0.001	0.094	0.015
		800/200	0.081	0.000	0.072	0.000
	Large	80/20	0.167	−0.001	0.159	−0.220
		160/40	0.165	−0.001	0.184	−0.023
		320/80	0.165	0.001	0.155	−0.003
		800/200	0.161	0.000	0.142	0.004

*DIF, Differential item functioning or measurement non-invariance; No difference, no factor mean difference; Difference, factor mean difference; Intercept, intercept factor mean; Slope, slope factor mean; Intercept d, intercept factor mean difference; Slope d, slope factor mean difference*.

## Study 2: second-order growth mixture modeling

### Method

#### Fitted models and simulation outcomes

The data generated in Study 1 were fitted to the second-order growth mixture models that allow heterogeneity in the measurement parameters at the first-order measurement model. The second-order part was specified identical to the GMM in Study 1. Instead of using observed mean composite scores for the indictors of the intercept and slope factors, a latent factor on which six items loaded was included at each occasion as shown in Figure 2. As in Study 1, we fitted one-, two-, and three-class models and decided the number of classes based on the fit criteria (lowest information criterion) applying the minimum class proportion 0.05 rule. Because two latent classes were generated in the population and SOGMM was a correctly specified model, two classes were expected to be selected across all simulation conditions. In addition, bias or relative bias in parameter estimates was evaluated. The parameters of interest included the differences between classes in the intercept and slope factor means and the size of noninvariance in the factor loadings and intercepts of two noninvariant items. The size of noninvariance was averaged across two items. It was hypothesized that SOGMM would yielded unbiased estimates of these parameters. Mplus version 7.3 (Muthén and Muthén, 2014) was used for SOGMM.

### Results

#### Class enumeration

The class enumeration rates of AIC, BIC, saBIC, and HBIC are presented in Tables 3, 4. Because population heterogeneity was simulated between two classes either in measurement parameters only (no factor mean difference conditions in Table 3) or in both measurement and structural parameters (factor mean difference conditions in Table 4), we hypothesized that SOGMM would identify two latent classes correctly. However, the correct enumeration rates varied depending on the fit criteria and simulation factors. First, when there were differences in both measurement and structural parameters with substantial factor mean differences, the BIC and HBIC almost always endorsed two classes correctly. However, when measurement noninvariance was the only source of population heterogeneity (i.e., lower class separation), the correct enumeration rates of BIC and HBIC deteriorated notably as sample size decreased and the noninvariance size was small. For example, the BIC was totally insensitive to the small noninvariance in the intercepts and selected one class across all replications. Under these conditions, the outperformance of HBIC over BIC was observed. On the other hand, the performance of saBIC was related more with sample size but less with the magnitude of noninvariance and class separation. Thus, as sample size increased, the correct enumeration rates of saBIC reached 100% with a few exceptions in the small intercept noninvariance only conditions. Interestingly, no salient difference was observed between small and large noninvariance conditions and also between no factor mean difference and factor mean difference conditions. For the AIC, the impact of sample size was observed only when the class separation was low (i.e., small noninvariance conditions without factor mean differences). When the class separation was sufficiently large (i.e., large noninvariance conditions even without factor mean differences), the overall performance of AIC was less affected by other simulation factors showing consistent enumeration rates. Of note is that as classes were separated more including factor mean differences, the AIC tended to over-extract latent classes more frequently.

**Table 3:** The class enumeration rates of second-order growth mixture modeling under the no factor mean difference conditions.

**DIF location**	**DIF size**	**Sample size**	**AIC**			**BIC**			**saBIC**			**HBIC**	
			**Number of latent classes**										
			**1**	**2**	**3**	**1**	**2^a^**	**1**		**2**	**3**	**1**	**2^b^**
Loading	Small	50/50	0.18	**0.77**	0.05	0.99	**0.01**	0.03		**0.80**	0.17	0.63	**0.37**
		100/100	0.03	**0.90**	0.07	0.90	**0.10**	0.03		**0.92**	0.05	0.52	**0.48**
		200/200	–	**0.93**	0.07	0.36	**0.64**	0.00		**0.99**	0.01	0.10	**0.90**
		500/500	–	**0.94**	0.06	–	**1.00**	–		**1.00**	–	–	**1.00**
	Large	50/50	–	**0.90**	0.10	–	**1.00**	–		**0.74**	0.26	–	**0.98** ^b^
		100/100	–	**0.90**	0.10	–	**1.00**	–		**0.93**	0.07	–	**1.00**
		200/200	–	**0.94**	0.06	–	**1.00**	–		**0.99**	0.01	–	**1.00**
		500/500	–	**0.95**	0.05	–	**1.00**	–		**1.00**	–	–	**1.00**
Intercept	Small	50/50	0.65	**0.31**	0.04	1.00	–	0.22		**0.66**	0.12	0.84	**0.15** ^b^
		100/100	0.59	**0.39**	0.02	1.00	–	0.66		**0.33**	0.01	0.92	**0.08**
		200/200	0.46	**0.51**	0.03	1.00	–	0.83		**0.16**	0.00	0.96	**0.04**
		500/500	0.08	**0.87**	0.04	1.00	**0.00**	0.70		**0.30**	–	0.95	**0.05**
	Large	50/50	0.02	**0.88**	0.09	0.80	**0.20**	0.00		**0.78**	0.22	0.29	**0.69** ^b^
		100/100	–	**0.92**	0.08	0.27	**0.73**	–		**0.95**	0.05	0.06	**0.94**
		200/200	–	**0.95**	0.05	0.00	**1.00**	–		**0.99**	0.01	–	**1.00**
		500/500	–	**0.97**	0.03	–	**1.00**	–		**1.00**	–	–	**1.00**
Loading	Small	80/20	0.46	**0.51**	0.03	1.00	**0.00**	0.12		**0.75**	–	0.77	**0.23**
		160/40	0.25	**0.71**	0.04	1.00	**0.00**	0.31		**0.67**	–	0.79	**0.21**
		320/80	0.06	**0.89**	0.05	0.95	**0.05**	0.21		**0.78**	0.01	0.54	**0.46**
		800/200	0.00	**0.95**	0.05	0.32	**0.68**	0.01		**0.99**	–	0.03	**0.97**
	Large	80/20	–	**0.93**	0.07	0.08	**0.92**	–		**0.75**	0.25	0.01	**0.99** ^b^
		160/40	–	**0.93**	0.07	–	**1.00**	–		**0.95**	0.05	–	**1.00** ^b^
		320/80	–	**0.92**	0.08	–	**1.00**	–		**1.00**	0.00	–	**1.00** ^b^
		800/200	–	**0.96**	0.04	–	**1.00**	–		**1.00**	–	–	**1.00**
Intercept	Small	80/20	0.72	**0.25**	0.03	1.00	–	0.23		**0.64**	0.13	0.85	**0.15** ^b^
		160/40	0.70	**0.29**	0.01	1.00	–	0.77		**0.23**	0.01	0.93	**0.07**
		320/80	0.68	**0.31**	0.01	1.00	–	0.94		**0.06**	–	0.94	**0.06**
		800/200	0.47	**0.50**	0.03	1.00	–	0.97		**0.03**	–	0.97	**0.03**
	Large	80/20	0.18	**0.76**	0.07	0.97	**0.03**	0.04		**0.78**	0.18	0.59	**0.41** ^b^
		160/40	0.02	**0.92**	0.06	0.88	**0.12**	0.02		**0.92**	0.05	0.33	**0.67** ^b^
		320/80	–	**0.94**	0.06	0.33	**0.67**	–		**0.99**	0.01	0.03	**0.97**
		800/200	–	**0.97**	0.03	–	**1.00**	–		**1.00**	–	–	**1.00**

*The hypothesized correct enumeration rates are in bold. DIF, Differential item functioning or measurement noninvariance; AIC, Akaike information criterion; BIC, Bayesian information criterion; saBIC, sample-size adjusted BIC; HBIC, hierarchical BIC. Due to rounding 0.00 means one or two replications out of 500*.

a *We compared one-, two-, and three-class models, but the number is not shown when the enumeration rates are zero across all conditions*.

b *The three-class model was selected with a small proportion*.

**Table 4:** The class enumeration rates of second-order growth mixture modeling under the factor mean difference conditions.

**DIF location**	**DIF size**	**Sample size**	**AIC**		**BIC**		**saBIC**		**HBIC**	
			**Number of latent classes**							
			**2**	**3**	**1**	**2^a^**	**2**	**3**	**1**	**2**
Loading	Small	50/50	**0.80**	0.20	0.00	**1.00**	**0.44**	0.56	–	**0.98** ^c^
		100/100	**0.82**	0.18	–	**1.00**	**0.88**	0.12	–	**1.00** ^c^
		200/200	**0.84**	0.16	–	**1.00**	**0.98**	0.02	–	**1.00**
		500/500	**0.95**	0.05	–	**1.00**	**1.00**	–	–	**1.00**
	Large	50/50	**0.84**	0.16	–	**1.00**	**0.48**	0.52	–	**0.96** ^c^
		100/100	**0.85**	0.15	–	**1.00**	**0.89**	0.11	–	**0.99** ^c^
		200/200	**0.89**	0.11	–	**1.00**	**0.99**	0.01	–	**1.00**
		500/500	**0.91**	0.09	–	**1.00**	**1.00**	–	–	**1.00**
Intercept	Small	50/50	**0.77** ^b^	0.21	0.59	**0.41**	**0.43** ^b^	0.56	0.09	**0.89** ^c^
		100/100	**0.78** ^b^	0.22	0.02	**0.98**	**0.84** ^b^	0.15	0.00	**1.00**
		200/200	**0.81** ^b^	0.19	0.00	**1.00**	**0.98** ^b^	0.02	0.00	**1.00**
		500/500	**0.94**	0.06	–	**1.00**	**1.00**	–	–	**1.00**
	Large	50/50	**0.77**	0.23	0.01	**0.99**	**0.40**	0.60	0.00	**0.98** ^c^
		100/100	**0.81**	0.19	–	**1.00**	**0.86**	0.14	–	**1.00** ^c^
		200/200	**0.85**	0.15	–	**1.00**	**0.99**	0.01	–	**1.00**
		500/500	**0.97**	0.03	–	**1.00**	**1.00**	–	–	**1.00**
Loading	Small	80/20	**0.80** ^b^	0.19	0.00	**1.00**	**0.52** ^b^	0.47	0.00	**0.99** ^c^
		160/40	**0.85**	0.15	–	**1.00**	**0.88**	0.12	–	**1.00** ^c^
		320/80	**0.83**	0.17	–	**1.00**	**0.99**	0.01	–	**1.00**
		800/200	**0.93**	0.07	–	**1.00**	**1.00**		–	**1.00**
	Large	80/20	**0.82**	0.18	–	**1.00**	**0.51**	0.49	–	**0.99** ^c^
		160/40	**0.86**	0.14	–	**1.00**	**0.89**	0.11	–	**1.00** ^c^
		320/80	**0.86**	0.14	–	**1.00**	**0.99**	0.01	–	**1.00**
		800/200	**0.92**	0.08	–	**1.00**	**1.00**	.	–	**1.00**
Intercept	Small	80/20	**0.77** ^b^	0.17	0.06	**0.94**	**0.48** ^b^	0.50	0.06	**0.94** ^c^
		160/40	**0.80** ^b^	0.17	0.05	**0.95**	**0.84** ^b^	0.12	0.04	**.96**
		320/80	**0.84** ^b^	0.15	0.01	**0.99**	**0.98** ^b^	0.02	0.01	**.99**
		800/200	**0.88**	0.12	–	**1.00**	**1.00**	–	–	**1.00**
	Large	80/20	**0.80**	0.20	0.00	**1.00**	**0.45**	0.55	0.00	**0.99** ^c^
		160/40	**0.84**	0.16	–	**1.00**	**0.88**	0.12	–	**1.00** ^c^
		320/80	**0.87**	0.13	–	**1.00**	**0.99**	0.01	–	**1.00**
		800/200	**0.88**	0.12	–	**1.00**	**1.00**	–	–	**1.00**

*The hypothesized correct enumeration rates are in bold. DIF, Differential item functioning or measurement noninvariance; AIC, Akaike information criterion; BIC, Bayesian information criterion; saBIC, sample-size adjusted BIC; HBIC, hierarchical BIC. Due to rounding 0.00 means one or two replications out of 500*.

a *We compared one-, two-, and three-class models, but the three-class model was not selected across all conditions*.

b *The one-class model was selected with a small proportion*.

c *The three-class model was selected with a small proportion*.

The impact of mixing proportion (balanced and unbalanced) was not very noticeable and inconsistent across simulation conditions. For example, the unbalanced conditions showed slightly lower correct enumeration rates of BIC when there was only measurement noninvariance at the measurement model. It is possibly related to the bias in noninvariance size. That is, noninvariance size was underestimated more in the unbalanced conditions (see bias and relative bias below). However, although not very noticeable, the opposite pattern was observed in the conditions of both measurement noninvariance and factor mean difference conditions. Overall, accurate class enumeration (and relatedly accurate parameter estimation) appeared more challenging when one class had a notably small sample size under low class separation, but when sample size and class separation became larger with both measurement noninvariance and factor mean difference, the impact of a small class was not observed. However, it should be replicated in future research. When two classes were identified, the mixing proportions were generally well recovered with about 50%, 50% for balanced conditions and about 80%, 20% for unbalanced conditions save the unbalanced conditions under small measurement noninvariance in which the mixing proportions turned out to be close to 50%, 50% as sample size became smaller.

#### Bias and relative bias

We examined the bias or relative bias of parameter estimates in terms of the size of noninvariance and factor mean differences when two latent classes were correctly identified. Because SOGMM was correctly specified, all parameter estimates were expected to be unbiased. As expected, the relative bias of factor loading and intercept noninvariance was negligible in most conditions when there were differences in both measurement parameters and factor means between classes. On the contrary, when measurement noninvariance was the only sources of population heterogeneity between classes, the noninvariance size was underestimated consistently across conditions as shown in Table 5 (left panel). The relative bias ranged from −0.788 to −0.010. The variability of relative bias was in general associated with sample size (the larger, the smaller), mixing proportion (smaller with the balanced proportions), and noninvariance size (smaller with large noninvariance). Note that under these conditions growth parameters were still unbiased. It appeared that the class specific measurement parameters (i.e., noninvariance) in the first order model were in general less accurately estimated than the class specific growth parameters (i.e., structural parameters) in the second order model when class separation was low. The estimation of the first (i.e., noninvariance size) improved with a larger sample and bigger separation. With respect to mixing proportions, the estimation could be less accurate with a disproportionately smaller class size in the unbalanced conditions when the total sample size was small (e.g., 20 when total *N* = 100)^1^. The underestimation of noninvariance size was possibly related to the lower enumeration rates under the measurement noninvariance only conditions (no factor mean difference) because in these conditions noninvariance was the only source of heterogeneity that separated two classes.

**Table 5:** The bias and relative bias of the parameter estimates in second-order growth mixture modeling.

**DIF location**	**DIF size**	**Sample size**	**No difference**	**Difference**	**No difference**		**Difference**	
			**Raw bias**	**Rel. bias**	**Raw bias**		**Rel. bias**	
			**DIF**	**DIF**	**Intercept**	**Slope**	**Intercept *d***	**Slope *d***
Loading	Small	50/50	−0.600	0.022	−0.039	0.121	−0.008	−0.020
		100/100	−0.515	0.008	−0.021	0.053	0.007	−0.013
		200/200	−0.403	−0.005	−0.005	0.027	0.000	−0.005
		500/500	−0.190	0.000	−0.003	0.007	−0.001	−0.005
	Large	50/50	−0.425	0.000	−0.020	0.027	−0.009	−0.023
		100/100	−0.321	0.000	−0.009	0.012	0.001	−0.003
		200/200	−0.184	−0.003	−0.002	0.008	0.000	0.000
		500/500	−0.018	0.003	−0.001	0.000	0.001	0.000
Intercept	Small	50/50	−0.157	−0.077	−0.129	0.230	−0.034	−0.095
		100/100	−0.327	0.000	−0.111	0.141	−0.018	−0.018
		200/200	−0.267	0.005	−0.060	0.095	−0.001	−0.005
		500/500	−0.173	0.000	−0.006	0.028	0.002	0.009
	Large	50/50	−0.310	−0.004	−0.026	0.049	0.002	0.010
		100/100	−0.231	−0.006	−0.012	0.014	0.000	0.000
		200/200	−0.091	−0.003	−0.001	0.005	0.004	0.000
		500/500	−0.010	0.008	0.004	0.001	−0.002	0.001
Loading	Small	80/20	−0.788	0.007	−0.105	0.210	0.013	−0.080
		160/40	−0.713	0.032	−0.041	0.119	0.002	−0.015
		320/80	−0.493	0.015	0.014	0.059	0.002	−0.003
		800/200	−0.240	−0.003	−0.004	0.012	0.002	−0.003
	Large	80/20	−0.456	0.003	−0.019	0.028	0.003	−0.005
		160/40	−0.450	0.006	−0.017	0.021	0.002	0.006
		320/80	−0.408	0.003	0.005	0.016	0.001	0.003
		800/200	−0.375	0.000	−0.003	0.005	0.001	0.001
Intercept	Small	80/20	−0.357	−0.142	−0.180	0.251	0.016	−0.143
		160/40	−0.395	−0.030	−0.122	0.210	−0.004	−0.005
		320/80	−0.317	−0.020	−0.100	0.154	0.004	0.006
		800/200	−0.328	−0.005	−0.074	0.073	0.001	−0.008
	Large	80/20	−0.463	−0.018	−0.091	0.100	0.012	−0.065
		160/40	−0.339	−0.003	−0.033	0.029	0.003	−0.013
		320/80	−0.304	−0.014	−0.003	0.013	0.004	−0.003
		800/200	−0.144	−0.003	0.001	0.003	0.001	−0.003

*DIF, Differential item functioning or measurement non-invariance; No difference, no factor mean difference; Difference, factor mean difference; Rel. bias, relative bias; Intercept, intercept factor mean; Slope, slope factor mean; Intercept d, intercept factor mean difference; Slope d, slope factor mean difference*.

The right panel of Table 5 presents the bias or relative bias of factor mean difference estimates between classes. Of note is that the bias or relative bias was estimated for the factor mean differences (not for the factor means). When there were factor mean differences, the estimated differences were generally unbiased regardless of simulation factors. The relative bias of the factor mean differences between classes was <0.05 across conditions except the smallest sample size conditions (*N* = 100; see the two columns of the last panel in Table 5). When there was no factor mean difference and two classes were different only due to measurement noninvariance, the estimated factor means generally showed no difference between classes (i.e., no bias) with large sample, but when sample size was small, bigger size of raw bias was observed (See the two columns of the middle panel in Table 5).

## Discussion

When researchers run GMM, it is a common practice to use composite scores of repeated measures to model the baseline performance and growth over time. This could be problematic when the measure does not have desirable psychometric properties because GMM does not allow evaluating measurement models. In this study we addressed one of these issues—measurement noninvariance. When there was measurement noninvariance between unknown groups, we investigated the impact of the ignored noninvariance on the performance of GMM, particularly, the accuracy of class enumeration and the parameter recovery. In addition, we examined the performance of SOGMM that incorporates measurement models and allows measurement noninvariance between latent classes.

First, we hypothesized that due to unmodeled noninvariance in items GMM would incorrectly identify two latent classes showing differences in factor means between classes when there was no difference in factor means. In Study 1, this hypothesis was not supported because the BIC and HBIC mostly selected a one-class model as a best-fitting model. However, this finding should not be interpreted as no impact of the ignored measurement noninvariance on the GMM class enumeration. Rather, it might indicate that overall, GMM is not very sensitive to a small degree of population heterogeneity. This was confirmed in the conditions with both measurement noninvariance and factor mean differences. Although the generated class separation (MD or mahalanobis distance = 2) in the factor mean differences was considered large, the BIC, for example, supported one class more often when sample size was 400 or less. Under these conditions the enumeration rates of BIC were associated with the location and size of noninvariance, which implies that the ignored measurement noninvariance affected the performance of GMM, specifically, the enumeration accuracy of BIC and HBIC.

Second, as hypothesized, the parameter estimates of GMM, namely, the intercept and slope factor means were biased regardless of simulation factors. The location, size, and direction of bias were directly related to the location, size, and direction of unmodeled measurement noninvariance. That is, we observed positive bias in the intercept factor when positive noninvariance in the item intercepts were ignored and negative bias in the slope factor when negative noninvariance in the item factor loadings were ignored. When the size of noninvariance was doubled, the size of bias was also doubled. This finding is consistent to what Kim and Willson (2014a) found with multiple group LGM. Because GMM yields biased parameter estimates even if the number of latent classes is correctly detected, GMM is not recommended in the presence of measurement noninvariance.

Third, with respect to SOGMM, our hypothesis about high accuracy of class enumeration of SOGMM was partly supported in Study 2 because class enumeration rates largely depended on class separation and sample size. When the class separation was large under the conditions of both measurement noninvariance and factor mean differences, the correct enumeration rates of BIC were almost 100% even with a very small sample size (i.e., 100). However, when the class separation was low (small noninvariance only) and sample size was small (400 or less), the correct enumeration rates of BIC dropped substantially (e.g., 0%). A previous simulation study (Lubke and Neale, 2008) that investigated the class enumeration rates in detecting measurement noninvariance also found that more parsimonious models (e.g., one-class model) were favored indicating no measurement noninvariance. The overall insensitivity of ICs to the presence of small measurement noninvariance between latent classes can be explained as relatively low class separation that measurement noninvariance created. The small noninvariance in the intercepts corresponded to MD = 1, which is considered as small class separation in the literature. It is widely recognized that class separation is greatly related to the accuracy of class enumeration (e.g., Henson et al., 2007; Tofighi and Enders, 2008; Chen et al., 2010).

Fourth, the hypothesis that SOGMM would yield unbiased estimates was also partly correct. As hypothesized, the intercept and slope factor means of SOGMM were generally unbiased. When sample size was very small, we observed some biased estimates of these parameters. On the other hand, the size of noninvariance in the intercepts and factor loadings were generally underestimated. This underestimation of noninvariance size might make it more difficult for ICs to detect the difference and be partly related to the low class enumeration accuracy when measurement noninvariance was the only source of population heterogeneity between classes.

With respect to information criteria, the findings in this study generally conform to those of previous studies. The BIC showed excellent performance in identifying the number of classes when class separation was large and sample size was large (Nylund et al., 2007; Lubke and Neale, 2008; Li et al., 2009). When both class separation was low and sample size was small, the BIC tended to under-extract latent classes (Kim et al., 2016). The HBIC showed similar or slightly better performance than the BIC. The outperformance of HBIC was prominent when sample size and class separation were small, which is consistent to the findings of previous studies (e.g., Zhao et al., 2015). It should be noted that the over-extraction of latent classes was also observed with the HBIC, which is possibly due to under-penalization of model complexity compared to the BIC although the over-extraction was not very serious in this study. The saBIC showed more consistent performance across simulation conditions, but its accuracy was lower compared to BIC and HBIC when these two ICs worked reasonably. The AIC seemed least affected by simulation factors usually showing consistent enumeration rates across simulation conditions and most sensitive to population heterogeneity in the extreme conditions (i.e., smallest sample size in this study under low class separation; e.g., Lukočienė and Vermunt, 2010; Lukočienė et al., 2010; Kim et al., 2016), but the performance of AIC was generally not optimal and also tended to over-extract latent classes (e.g., Bozdogan, 1987; Nylund et al., 2007; Tein et al., 2013). As explained in previous studies (Henson et al., 2007; Kim et al., 2015, 2016), the BIC uses the natural logarithm of sample size multiplied by the number of free parameters (*k*) to penalize for model complexity. The penalty of BIC on additionally estimated parameters (i.e., additional latent class) is more severe than that of AIC (2^*^k). Thus, when class separation is low, a complex model with more parameters from additional latent classes may not be favored with the BIC due to too severe penalty on model complexity relative to small differences between latent classes. Under these circumstances, the AIC as well as HBIC generally outperformed the BIC. Also the AIC is not supposed to be affected by sample size as much as the other ICs that include sample size in their computations.

Taken all together, when sample size is large (over 400 or 1,000 in this study) or class separation is expected to be large, the BIC or HBIC is recommended in GMM. When sample size is 400 or less and class separation is expected to be low, the saBIC seems a better choice in GMM. In SOGMM, if class separation is substantially large (MD = 2 or larger), the BIC or HBIC can be considered for class enumeration regardless of sample size. However, similar to GMM, when class separation is expected to be low and sample size is 400 or less, the saBIC is more recommended than the BIC and HBIC in determining the number of latent classes. The AIC could be a choice only when sample size is extremely small (100), but the mixture modeling is not recommended with this small sample.

Based on the findings in this study, it can be said that overall, GMM and SOGMM require large sample to correctly identify the number of classes and yield unbiased parameter estimates (Tueller and Lubke, 2010; Depaoli, 2013; Li and Harring, 2016). Vermunt (2010) noted that sample size 500 can be considered small for correct class enumeration especially under poor class separation, which was observed in this study particularly with the BIC. Even when the model is correctly specified as demonstrated in Study 2 with SOGMM, latent classes are not expected to be properly detected with small samples. Even when the number of latent classes is correctly identified, the parameter estimates could be substantially biased. Therefore, researchers interested in GMM or SOGMM should consider a large sample. This study also confirmed that class separation and sample size are generally major factors related to the class enumeration accuracy, which was consistently shown in the mixture modeling literature (e.g., Dias, 2004; Henson et al., 2007; Lubke and Neale, 2008; Tofighi and Enders, 2008; Chen et al., 2010).

We recommend SOGMM over GMM whenever possible for two major reasons. First, across all simulation conditions SOGMM produced unbiased estimates of growth trajectory parameters which are generally the focal interest of growth analysis in psychological research. SOGMM is advantageous because it includes measurement models of repeated measures that take into account measurement error and allows heterogeneity in measurement parameters between unknown groups. If there are differences in measurement models between potential groups, the differences can be captured by heterogeneous latent classes in SOGMM. As illustrated in this study, when the heterogeneity in measurement models is ignored and GMM is run, the parameter estimates of GMM are expected to be biased to the degree of the size of ignored differences. For example, developmental psychologists may observe an inflated difference between increasing and decreasing trajectory classes in terms of depressive symptoms. Or they may observe a smaller difference between them due to biased trajectory estimates. It was also observed that the ignored measurement noninvariance impacted on the enumeration rates of BIC and HBIC. Of note is that measurement invariance across latent classes cannot be tested separately using longitudinal common factor models because latent classes are unknown in advance. Thus, SOGMM is more imperative when researchers are interested in unknown clustering of growth trajectories. Second, SOGMM generally showed more accurate class enumeration possibly because it could directly detect any differences in the measurement models as well as in the growth model.

Finally, it should be kept in mind that some simulation factors were manipulated for the purpose of the study and hence generalization of the results beyond the simulation settings should be done with caution. For example, to highlight the impact of ignored measurement noninvariance between latent classes, we assumed measurement invariance over time. In reality, this assumption is not guaranteed and researchers should test and establish the temporal measurement invariance. Another assumption we made for the simplicity of discussion is no error correlation over time, but this assumption is less likely to be met with real data. In the presence of error correlation over time, the correct class enumeration is possibly more challenging because class separation becomes lower. Future research is called for the impact of different types of error structures on the performance of GMM and SOGMM.

## Author contributions

All authors (EK, YW) contribute to the paper substantially and agree to be accountable for the content of the work.

### Conflict of interest statement

The authors declare that the research was conducted in the absence of any commercial or financial relationships that could be construed as a potential conflict of interest. The reviewer DM and handling Editor declared their shared affiliation.

## Footnotes

^1^It should be noted that the accuracy of class membership assignment appeared not related to the underestimation of noninvariance size because (a) the growth parameters were unbiased under the same conditions, and (b) we did not observe an apparent association between the assignment accuracy and bias. For example, under the unbalanced conditions in which the underestimation was larger, the assignment accuracy was even slightly higher compared to the balanced conditions.
