# Supplementary material for: Class Enumeration and Parameter Recovery of Growth Mixture Modeling and Second-Order Growth Mixture Modeling in the Presence of Measurement Noninvariance between Latent Classes
Source: Front Psychol. 2017 Sep 5;8:1499. doi: 10.3389/fpsyg.2017.01499 (PMC5591846; doi:10.3389/fpsyg.2017.01499)
Supplement: Supplementary file 1 [file DataSheet1.docx]

**Appendix**

In second-order growth mixture modeling (SOGMM), the expectation (particularly, a mean vector) of a continuous observed variable ***Y*** is estimated for latent class *c* as (Equation 10):

$$\boldsymbol{\mu}_{c}=\boldsymbol{\nu}_{c}+\boldsymbol{\Lambda}_{c}\boldsymbol{\Gamma}_{\boldsymbol{c}}\boldsymbol{\kappa}_{\boldsymbol{c}}\boldsymbol{.}$$

From this equation, we can derive:

$$\boldsymbol{\kappa}_{c}\boldsymbol{=}{\boldsymbol{\Gamma}_{\boldsymbol{c}}}^{-1}{\boldsymbol{\Lambda}_{c}}^{-1}\boldsymbol{(}\boldsymbol{\mu}_{c}-\boldsymbol{\nu}_{c})\boldsymbol{,}$$

For simplicity, we assume a linear growth model for two latent classes and $\boldsymbol{\Gamma}_{\boldsymbol{c}}$ is simplified to $\boldsymbol{\Gamma}$. When measurement invariance holds, that is, $\boldsymbol{\nu}_{c}=\boldsymbol{\nu}; \boldsymbol{\Lambda}_{c}=\boldsymbol{\Lambda}$, the expected value specific to a latent class $\boldsymbol{\mu}_{c}$ is a function of class-specific latent factor means $\boldsymbol{\kappa}_{\boldsymbol{c}}$ (or vice versa) as shown in the equation below:

$$\boldsymbol{\kappa}_{c}\boldsymbol{=}\boldsymbol{\Gamma}^{-1}\boldsymbol{\Lambda}^{-1}\boldsymbol{(}\boldsymbol{\mu}_{c}-\boldsymbol{\nu})\boldsymbol{,}$$

If measurement invariance does not hold, the relation between the observed means and the factor means also depends on measurement parameters specific to a latent class ($\boldsymbol{\nu}_{c}$ and $\boldsymbol{\Lambda}_{c}$).

$$\boldsymbol{\kappa}_{c}\boldsymbol{=}\boldsymbol{\Gamma}^{-1}{\boldsymbol{\Lambda}_{c}}^{-1}\boldsymbol{(}\boldsymbol{\mu}_{c}-\boldsymbol{\nu}_{c})$$

In this case, if measurement invariance is falsely imposed, the estimates of latent factor means can be biased depending on the location and the direction of noninvariance. We illustrate how measurement noninvariance ($\boldsymbol{\nu}_{c}$ and $\boldsymbol{\Lambda}_{c}$) makes an impact on the estimates of factor means in four different scenarios using SOGMM.

**Case 1. Intercept noninvariance (**$\boldsymbol{\nu}_{\boldsymbol{c}}$**) with no factor mean difference (**$\boldsymbol{\kappa}_{\boldsymbol{c}}\boldsymbol{=\kappa}$**)**

Suppose a single factor measured repeatedly over three time points with two observed variables (total six observed variables). Assume there are two subpopulations (*c* = 1, 2). Let $\boldsymbol{\Gamma},\boldsymbol{\Lambda}, \boldsymbol{\kappa}$ be

$$\boldsymbol{\Gamma}=\left[ \begin{matrix} 1 & 0 \\ 1 & 1 \\ 1 & 2 \end{matrix} \right], \boldsymbol{\Lambda}=\left[ \begin{aligned} \begin{matrix} 1 & 0 & 0 \\ 1 & 0 & 0 \\ 0 & 1 & 0 \end{matrix} \\ \begin{matrix} 0 & 1 & 0 \\ 0 & 0 & 1 \\ 0 & 0 & 1 \end{matrix} \end{aligned} \right],\boldsymbol{\kappa}=\left[ \begin{aligned} 0 \\ 1 \end{aligned} \right]$$

and $\boldsymbol{\nu}$ be class specific as

$$\boldsymbol{\nu}_{1}=\left[ \begin{aligned} 0 \\ 0 \\ 0 \\ 0 \\ 0 \\ 0 \end{aligned} \right], \boldsymbol{\nu}_{2}=\left[ \begin{aligned} 0 \\ 0.6 \\ 0 \\ 0.6 \\ 0 \\ 0.6 \end{aligned} \right].$$

Then,

$$\boldsymbol{\mu}_{1}=\left[ \begin{aligned} 0 \\ 0 \\ 0 \\ 0 \\ 0 \\ 0 \end{aligned} \right]+\left[ \begin{aligned} \begin{matrix} 1 & 0 & 0 \\ 1 & 0 & 0 \\ 0 & 1 & 0 \end{matrix} \\ \begin{matrix} 0 & 1 & 0 \\ 0 & 0 & 1 \\ 0 & 0 & 1 \end{matrix} \end{aligned} \right]*\left[ \begin{matrix} 1 & 0 \\ 1 & 1 \\ 1 & 2 \end{matrix} \right]*\left[ \begin{aligned} 0 \\ 1 \end{aligned} \right]=\left[ \begin{aligned} 0 \\ 0 \\ 1 \\ 1 \\ 2 \\ 2 \end{aligned} \right],$$

$$\boldsymbol{\mu}_{2}=\left[ \begin{aligned} 0 \\ 0.6 \\ 0 \\ 0.6 \\ 0 \\ 0.6 \end{aligned} \right]+\left[ \begin{aligned} \begin{matrix} 1 & 0 & 0 \\ 1 & 0 & 0 \\ 0 & 1 & 0 \end{matrix} \\ \begin{matrix} 0 & 1 & 0 \\ 0 & 0 & 1 \\ 0 & 0 & 1 \end{matrix} \end{aligned} \right]*\left[ \begin{matrix} 1 & 0 \\ 1 & 1 \\ 1 & 2 \end{matrix} \right]*\left[ \begin{aligned} 0 \\ 1 \end{aligned} \right]=\left[ \begin{aligned} 0 \\ 0.6 \\ 1 \\ 1.6 \\ 2 \\ 2.6 \end{aligned} \right].$$

Because we illustrate how measurement noninvariance makes an impact on the estimates of factor means, we derive the factor means for each class given $\boldsymbol{\mu}_{1}$ and $\boldsymbol{\mu}_{2}$ when intercept noninvariance between classes is (a) correctly modeled and (b) falsely ignored. First, when intercept noninvariance is correctly specified,

$$\boldsymbol{\kappa}_{1}\boldsymbol{=}\boldsymbol{\Gamma}^{-1}\boldsymbol{\Lambda}^{-1}\left( \boldsymbol{\mu}_{1}-\boldsymbol{\nu}_{1} \right)=\left[ \begin{matrix} 1 & 0 \\ 1 & 1 \\ 1 & 2 \end{matrix} \right]^{\boldsymbol{-1}}\boldsymbol{*}\left[ \begin{aligned} \begin{matrix} 1 & 0 & 0 \\ 1 & 0 & 0 \\ 0 & 1 & 0 \end{matrix} \\ \begin{matrix} 0 & 1 & 0 \\ 0 & 0 & 1 \\ 0 & 0 & 1 \end{matrix} \end{aligned} \right]^{\boldsymbol{-1}}\boldsymbol{*}\left( \left[ \begin{aligned} 0 \\ 0 \\ 1 \\ 1 \\ 2 \\ 2 \end{aligned} \right]\boldsymbol{-}\left[ \begin{aligned} 0 \\ 0 \\ 0 \\ 0 \\ 0 \\ 0 \end{aligned} \right] \right)\boldsymbol{=}\left[ \begin{aligned} 0 \\ 1 \end{aligned} \right],$$

$$\boldsymbol{\kappa}_{2}\boldsymbol{=}\boldsymbol{\Gamma}^{-1}\boldsymbol{\Lambda}^{-1}\left( \boldsymbol{\mu}_{2}-\boldsymbol{\nu}_{2} \right)=\left[ \begin{matrix} 1 & 0 \\ 1 & 1 \\ 1 & 2 \end{matrix} \right]^{\boldsymbol{-1}}\boldsymbol{*}\left[ \begin{aligned} \begin{matrix} 1 & 0 & 0 \\ 1 & 0 & 0 \\ 0 & 1 & 0 \end{matrix} \\ \begin{matrix} 0 & 1 & 0 \\ 0 & 0 & 1 \\ 0 & 0 & 1 \end{matrix} \end{aligned} \right]^{\boldsymbol{-1}}\boldsymbol{*}\left( \left[ \begin{aligned} 0 \\ 0.6 \\ 1 \\ 1.6 \\ 2 \\ 2.6 \end{aligned} \right]\boldsymbol{-}\left[ \begin{aligned} 0 \\ 0.6 \\ 0 \\ 0.6 \\ 0 \\ 0.6 \end{aligned} \right] \right)\boldsymbol{=}\left[ \begin{aligned} 0 \\ 1 \end{aligned} \right].$$

This illustrates that $\boldsymbol{\kappa}_{1}\boldsymbol{=}\boldsymbol{\kappa}_{2}\boldsymbol{=\kappa}$ as data were generated (i.e., no factor mean difference between classes) when intercept noninvariance is correctly modeled.

Suppose intercept invariance is assumed, that is, $\boldsymbol{\nu}_{1}=\boldsymbol{\nu}_{2}= \boldsymbol{\nu}$when $\boldsymbol{\nu}_{1}\neq\boldsymbol{\nu}_{2}$. Let the invariance assumed $\boldsymbol{\nu}$ be

$$\boldsymbol{\nu}=\left[ \begin{aligned} 0 \\ 0.3 \\ 0 \\ 0.3 \\ 0 \\ 0.3 \end{aligned} \right]$$

for both classes and estimate the factor means for each class.

$$\boldsymbol{\kappa}_{1}\boldsymbol{=}\boldsymbol{\Gamma}^{-1}\boldsymbol{\Lambda}^{-1}\left( \boldsymbol{\mu}_{1}-\boldsymbol{\nu} \right)=\left[ \begin{matrix} 1 & 0 \\ 1 & 1 \\ 1 & 2 \end{matrix} \right]^{\boldsymbol{-1}}\boldsymbol{*}\left[ \begin{aligned} \begin{matrix} 1 & 0 & 0 \\ 1 & 0 & 0 \\ 0 & 1 & 0 \end{matrix} \\ \begin{matrix} 0 & 1 & 0 \\ 0 & 0 & 1 \\ 0 & 0 & 1 \end{matrix} \end{aligned} \right]^{\boldsymbol{-1}}\boldsymbol{*}\left( \left[ \begin{aligned} 0 \\ 0 \\ 1 \\ 1 \\ 2 \\ 2 \end{aligned} \right]\boldsymbol{-}\left[ \begin{aligned} 0 \\ 0.3 \\ 0 \\ 0.3 \\ 0 \\ 0.3 \end{aligned} \right] \right)\boldsymbol{=}\left[ \begin{aligned} -0.15 \\ 1 \end{aligned} \right],$$

$$\boldsymbol{\kappa}_{2}\boldsymbol{=}\boldsymbol{\Gamma}^{-1}\boldsymbol{\Lambda}^{\boldsymbol{-1}}\left( \boldsymbol{\mu}_{2}-\boldsymbol{\nu} \right)=\left[ \begin{matrix} 1 & 0 \\ 1 & 1 \\ 1 & 2 \end{matrix} \right]^{\boldsymbol{-1}}\boldsymbol{*}\left[ \begin{aligned} \begin{matrix} 1 & 0 & 0 \\ 1 & 0 & 0 \\ 0 & 1 & 0 \end{matrix} \\ \begin{matrix} 0 & 1 & 0 \\ 0 & 0 & 1 \\ 0 & 0 & 1 \end{matrix} \end{aligned} \right]^{\boldsymbol{-1}}\boldsymbol{*}\left( \left[ \begin{aligned} 0 \\ 0.6 \\ 1 \\ 1.6 \\ 2 \\ 2.6 \end{aligned} \right]\boldsymbol{-}\left[ \begin{aligned} 0 \\ 0.3 \\ 0 \\ 0.3 \\ 0 \\ 0.3 \end{aligned} \right] \right)\boldsymbol{=}\left[ \begin{aligned} 0.15 \\ 1 \end{aligned} \right].$$

Because intercept values are subtracted more for class 1 and less for class 2 than they should be due to ignored intercept noninvariance, the intercept factor mean is underestimated for class 1 (-0.15 instead of 0) and overestimated for class 2 (0.15). Overall, the intercept factor mean difference is observed when there is no difference. On the other hand, the slope factor means are intact showing no difference between classes (1) as generated.

**Case 2. Factor loading noninvariance (**$\boldsymbol{\Lambda}_{\boldsymbol{c}}$**) with no factor mean difference (**$\boldsymbol{\kappa}_{\boldsymbol{c}}\boldsymbol{=\kappa}$**)**

If

$$\boldsymbol{\Lambda}_{1}=\left[ \begin{aligned} \begin{matrix} 1 & 0 & 0 \\ 1 & 0 & 0 \\ 0 & 1 & 0 \end{matrix} \\ \begin{matrix} 0 & 1 & 0 \\ 0 & 0 & 1 \\ 0 & 0 & 1 \end{matrix} \end{aligned} \right], \boldsymbol{\Lambda}_{2}=\left[ \begin{aligned} 1 \\ 0.6 \\ 0 \\ 0 \\ 0 \\ 0 \end{aligned} \begin{aligned} 0 \\ 0 \\ 1 \\ 0.6 \\ 0 \\ 0 \end{aligned} \begin{aligned} 0 \\ 0 \\ 0 \\ 0 \\ 1 \\ 0.6 \end{aligned} \right], \boldsymbol{\nu}_{1}=\boldsymbol{\nu}_{2}=\boldsymbol{\nu}=\left[ \begin{aligned} 0 \\ 0 \\ 0 \\ 0 \\ 0 \\ 0 \end{aligned} \right]$$

and the other terms remain the same as in Case 1, the observed means are computed as:

$$\boldsymbol{\mu}_{1}=\left[ \begin{aligned} 0 \\ 0 \\ 0 \\ 0 \\ 0 \\ 0 \end{aligned} \right]+\left[ \begin{aligned} \begin{matrix} 1 & 0 & 0 \\ 1 & 0 & 0 \\ 0 & 1 & 0 \end{matrix} \\ \begin{matrix} 0 & 1 & 0 \\ 0 & 0 & 1 \\ 0 & 0 & 1 \end{matrix} \end{aligned} \right]*\left[ \begin{matrix} 1 & 0 \\ 1 & 1 \\ 1 & 2 \end{matrix} \right]*\left[ \begin{aligned} 0 \\ 1 \end{aligned} \right]=\left[ \begin{aligned} 0 \\ 0 \\ 1 \\ 1 \\ 2 \\ 2 \end{aligned} \right],$$

$$\boldsymbol{\mu}_{2}=\left[ \begin{aligned} 0 \\ 0 \\ 0 \\ 0 \\ 0 \\ 0 \end{aligned} \right]+\left[ \begin{aligned} 1 \\ 0.6 \\ 0 \\ 0 \\ 0 \\ 0 \end{aligned} \begin{aligned} 0 \\ 0 \\ 1 \\ 0.6 \\ 0 \\ 0 \end{aligned} \begin{aligned} 0 \\ 0 \\ 0 \\ 0 \\ 1 \\ 0.6 \end{aligned} \right]*\left[ \begin{matrix} 1 & 0 \\ 1 & 1 \\ 1 & 2 \end{matrix} \right]*\left[ \begin{aligned} 0 \\ 1 \end{aligned} \right]=\left[ \begin{aligned} 0 \\ 0 \\ 1 \\ 0.6 \\ 2 \\ 1.2 \end{aligned} \right].$$

When measurement noninvariance in factor loadings is correctly modeled, the factor means given $\boldsymbol{\mu}_{1}$ and $\boldsymbol{\mu}_{2}$ are correctly derived as $\boldsymbol{\kappa}_{\boldsymbol{1}}\boldsymbol{=}\boldsymbol{\kappa}_{\boldsymbol{2}}\boldsymbol{=\kappa}$ as shown below:

$$\boldsymbol{\kappa}_{1}\boldsymbol{=}\boldsymbol{\Gamma}^{-1}{\boldsymbol{\Lambda}_{\boldsymbol{1}}}^{-1}\left( \boldsymbol{\mu}_{1}-\boldsymbol{\nu} \right)=\left[ \begin{matrix} 1 & 0 \\ 1 & 1 \\ 1 & 2 \end{matrix} \right]^{\boldsymbol{-1}}\boldsymbol{*}\left[ \begin{aligned} \begin{matrix} 1 & 0 & 0 \\ 1 & 0 & 0 \\ 0 & 1 & 0 \end{matrix} \\ \begin{matrix} 0 & 1 & 0 \\ 0 & 0 & 1 \\ 0 & 0 & 1 \end{matrix} \end{aligned} \right]^{\boldsymbol{-1}}\boldsymbol{*}\left( \left[ \begin{aligned} 0 \\ 0 \\ 1 \\ 1 \\ 2 \\ 2 \end{aligned} \right]\boldsymbol{-}\left[ \begin{aligned} 0 \\ 0 \\ 0 \\ 0 \\ 0 \\ 0 \end{aligned} \right] \right)\boldsymbol{=}\left[ \begin{aligned} 0 \\ 1 \end{aligned} \right],$$

$$\boldsymbol{\kappa}_{2}\boldsymbol{=}\boldsymbol{\Gamma}^{-1}{\boldsymbol{\Lambda}_{\boldsymbol{2}}}^{-1}\left( \boldsymbol{\mu}_{2}-\boldsymbol{\nu} \right)=\left[ \begin{matrix} 1 & 0 \\ 1 & 1 \\ 1 & 2 \end{matrix} \right]^{\boldsymbol{-1}}\boldsymbol{*}\left[ \begin{aligned} 1 \\ 0.6 \\ 0 \\ 0 \\ 0 \\ 0 \end{aligned} \begin{aligned} 0 \\ 0 \\ 1 \\ 0.6 \\ 0 \\ 0 \end{aligned} \begin{aligned} 0 \\ 0 \\ 0 \\ 0 \\ 1 \\ 0.6 \end{aligned} \right]^{\boldsymbol{-1}}\boldsymbol{*}\left( \left[ \begin{aligned} 0 \\ 0 \\ 1 \\ 0.6 \\ 2 \\ 1.2 \end{aligned} \right]\boldsymbol{-}\left[ \begin{aligned} 0 \\ 0 \\ 0 \\ 0 \\ 0 \\ 0 \end{aligned} \right] \right)\boldsymbol{=}\left[ \begin{aligned} 0 \\ 1 \end{aligned} \right].$$

When $\boldsymbol{\Lambda}_{1}\neq\boldsymbol{\Lambda}_{2}$, but factor loading invariance is assumed, that is, $\boldsymbol{\Lambda}_{1}=\boldsymbol{\Lambda}_{2}= \boldsymbol{\Lambda}$, let the invariance assumed $\boldsymbol{\Lambda}$ be

$$\boldsymbol{\Lambda}=\left[ \begin{aligned} 1 \\ 0.8 \\ 0 \\ 0 \\ 0 \\ 0 \end{aligned} \begin{aligned} 0 \\ 0 \\ 1 \\ 0.8 \\ 0 \\ 0 \end{aligned} \begin{aligned} 0 \\ 0 \\ 0 \\ 0 \\ 1 \\ 0.8 \end{aligned} \right].$$

Then,

$$\boldsymbol{\kappa}_{1}\boldsymbol{=}\boldsymbol{\Gamma}^{-1}\boldsymbol{\Lambda}^{-1}\left( \boldsymbol{\mu}_{1}-\boldsymbol{\nu} \right)=\left[ \begin{matrix} 1 & 0 \\ 1 & 1 \\ 1 & 2 \end{matrix} \right]^{\boldsymbol{-1}}\boldsymbol{*}\left[ \begin{aligned} 1 \\ 0.8 \\ 0 \\ 0 \\ 0 \\ 0 \end{aligned} \begin{aligned} 0 \\ 0 \\ 1 \\ 0.8 \\ 0 \\ 0 \end{aligned} \begin{aligned} 0 \\ 0 \\ 0 \\ 0 \\ 1 \\ 0.8 \end{aligned} \right]^{\boldsymbol{-1}}\boldsymbol{*}\left( \left[ \begin{aligned} 0 \\ 0 \\ 1 \\ 1 \\ 2 \\ 2 \end{aligned} \right]\boldsymbol{-}\left[ \begin{aligned} 0 \\ 0 \\ 0 \\ 0 \\ 0 \\ 0 \end{aligned} \right] \right)\boldsymbol{=}\left[ \begin{aligned} 0 \\ 1.1 \end{aligned} \right],$$

$$\boldsymbol{\kappa}_{2}\boldsymbol{=}\boldsymbol{\Gamma}^{-1}\boldsymbol{\Lambda}^{-1}\left( \boldsymbol{\mu}_{2}-\boldsymbol{\nu} \right)=\left[ \begin{matrix} 1 & 0 \\ 1 & 1 \\ 1 & 2 \end{matrix} \right]^{\boldsymbol{-1}}\boldsymbol{*}\left[ \begin{aligned} 1 \\ 0.8 \\ 0 \\ 0 \\ 0 \\ 0 \end{aligned} \begin{aligned} 0 \\ 0 \\ 1 \\ 0.8 \\ 0 \\ 0 \end{aligned} \begin{aligned} 0 \\ 0 \\ 0 \\ 0 \\ 1 \\ 0.8 \end{aligned} \right]^{\boldsymbol{-1}}\boldsymbol{*}\left( \left[ \begin{aligned} 0 \\ 0 \\ 1 \\ 0.6 \\ 2 \\ 1.2 \end{aligned} \right]\boldsymbol{-}\left[ \begin{aligned} 0 \\ 0 \\ 0 \\ 0 \\ 0 \\ 0 \end{aligned} \right] \right)\boldsymbol{=}\left[ \begin{aligned} 0 \\ 0.9 \end{aligned} \right].$$

It is illustrated that the slope factor mean is overestimated for class 1 with higher factor loadings and underestimated for class 2 with lower factor loadings while the intercept factor means remain the same between classes.

If factor loadings of class 2 are generated higher for noninvariance, the slope factor mean of class 2 will be overestimated whereas that of class 1 will be underestimated.

**Case 3. Positive pairing: A latent class with higher intercepts associated with higher factor means**

This is the case in which both intercepts and factor means are noninvariant ($\boldsymbol{\nu}_{c}$ and $\boldsymbol{\kappa}_{c}$). We use the same setting in Case 1 except that

$$\boldsymbol{\kappa}_{1}=\left[ \begin{aligned} 0 \\ 1 \end{aligned} \right], \boldsymbol{\kappa}_{2}=\left[ \begin{aligned} 1.4 \\ 1.4 \end{aligned} \right].$$

Thus, the expected means of six observed variables for each class are

$$\boldsymbol{\mu}_{1}=\left[ \begin{aligned} 0 \\ 0 \\ 1 \\ 1 \\ 2 \\ 2 \end{aligned} \right], \boldsymbol{\mu}_{2}=\left[ \begin{aligned} 1.4 \\ 2 \\ 2.8 \\ 3.4 \\ 4.2 \\ 4.8 \end{aligned} \right].$$

When noninvariance in intercepts is correctly specified, the factor means of each class are estimated as:

$$\boldsymbol{\kappa}_{1}\boldsymbol{=}\boldsymbol{\Gamma}^{-1}\boldsymbol{\Lambda}^{-1}\left( \boldsymbol{\mu}_{1}-\boldsymbol{\nu}_{1} \right)=\left[ \begin{matrix} 1 & 0 \\ 1 & 1 \\ 1 & 2 \end{matrix} \right]^{\boldsymbol{-1}}\boldsymbol{*}\left[ \begin{aligned} \begin{matrix} 1 & 0 & 0 \\ 1 & 0 & 0 \\ 0 & 1 & 0 \end{matrix} \\ \begin{matrix} 0 & 1 & 0 \\ 0 & 0 & 1 \\ 0 & 0 & 1 \end{matrix} \end{aligned} \right]^{\boldsymbol{-1}}\boldsymbol{*}\left( \left[ \begin{aligned} 0 \\ 0 \\ 1 \\ 1 \\ 2 \\ 2 \end{aligned} \right]\boldsymbol{-}\left[ \begin{aligned} 0 \\ 0 \\ 0 \\ 0 \\ 0 \\ 0 \end{aligned} \right] \right)\boldsymbol{=}\left[ \begin{aligned} 0 \\ 1 \end{aligned} \right],$$

$$\boldsymbol{\kappa}_{2}\boldsymbol{=}\boldsymbol{\Gamma}^{-1}\boldsymbol{\Lambda}^{-1}\left( \boldsymbol{\mu}_{2}-\boldsymbol{\nu}_{2} \right)=\left[ \begin{matrix} 1 & 0 \\ 1 & 1 \\ 1 & 2 \end{matrix} \right]^{\boldsymbol{-1}}\boldsymbol{*}\left[ \begin{aligned} \begin{matrix} 1 & 0 & 0 \\ 1 & 0 & 0 \\ 0 & 1 & 0 \end{matrix} \\ \begin{matrix} 0 & 1 & 0 \\ 0 & 0 & 1 \\ 0 & 0 & 1 \end{matrix} \end{aligned} \right]^{\boldsymbol{-1}}\boldsymbol{*}\left( \left[ \begin{aligned} 1.4 \\ 2 \\ 2.8 \\ 3.4 \\ 4.2 \\ 4.8 \end{aligned} \right]\boldsymbol{-}\left[ \begin{aligned} 0 \\ 0.6 \\ 0 \\ 0.6 \\ 0 \\ 0.6 \end{aligned} \right] \right)\boldsymbol{=}\left[ \begin{aligned} 1.4 \\ 1.4 \end{aligned} \right].$$

However, if we assume measurement invariance and apply a single set of intercepts ($\boldsymbol{\nu}'=\left[ 0 0.3 0 0.3 0 0.3 \right]$) instead of class specific intercepts ($\boldsymbol{\nu}_{1}$ and $\boldsymbol{\nu}_{2}$),

$$\boldsymbol{\kappa}_{1}\boldsymbol{=}\boldsymbol{\Gamma}^{-1}\boldsymbol{\Lambda}^{-1}\left( \boldsymbol{\mu}_{1}-\boldsymbol{\nu} \right)=\left[ \begin{matrix} 1 & 0 \\ 1 & 1 \\ 1 & 2 \end{matrix} \right]^{\boldsymbol{-1}}\boldsymbol{*}\left[ \begin{aligned} \begin{matrix} 1 & 0 & 0 \\ 1 & 0 & 0 \\ 0 & 1 & 0 \end{matrix} \\ \begin{matrix} 0 & 1 & 0 \\ 0 & 0 & 1 \\ 0 & 0 & 1 \end{matrix} \end{aligned} \right]^{\boldsymbol{-1}}\boldsymbol{*}\left( \left[ \begin{aligned} 0 \\ 0 \\ 1 \\ 1 \\ 2 \\ 2 \end{aligned} \right]\boldsymbol{-}\left[ \begin{aligned} 0 \\ 0.3 \\ 0 \\ 0.3 \\ 0 \\ 0.3 \end{aligned} \right] \right)\boldsymbol{=}\left[ \begin{aligned} -0.15 \\ 1 \end{aligned} \right],$$

$$\boldsymbol{\kappa}_{2}\boldsymbol{=}\boldsymbol{\Gamma}^{-1}\boldsymbol{\Lambda}^{-1}\left( \boldsymbol{\mu}_{2}-\boldsymbol{\nu} \right)=\left[ \begin{matrix} 1 & 0 \\ 1 & 1 \\ 1 & 2 \end{matrix} \right]^{\boldsymbol{-1}}\boldsymbol{*}\left[ \begin{aligned} \begin{matrix} 1 & 0 & 0 \\ 1 & 0 & 0 \\ 0 & 1 & 0 \end{matrix} \\ \begin{matrix} 0 & 1 & 0 \\ 0 & 0 & 1 \\ 0 & 0 & 1 \end{matrix} \end{aligned} \right]^{\boldsymbol{-1}}\boldsymbol{*}\left( \left[ \begin{aligned} 1.4 \\ 2 \\ 2.8 \\ 3.4 \\ 4.2 \\ 4.8 \end{aligned} \right]\boldsymbol{-}\left[ \begin{aligned} 0 \\ 0.3 \\ 0 \\ 0.3 \\ 0 \\ 0.3 \end{aligned} \right] \right)\boldsymbol{=}\left[ \begin{aligned} 1.55 \\ 1.4 \end{aligned} \right].$$

As illustrated here, the intercept factor mean is negatively biased for class 1 with lower factor means and positively biased for class 2 with higher factor means, which leads to the overestimation of the intercept factor mean difference between classes. The impact of intercept noninvariance on the slope factor means is null.

Similarly, when a latent class with higher intercepts is paired with lower factor means (negative pairing), the intercept factor mean of this class will be overestimated whereas that of the class with higher factor means will be underestimated, which makes the intercept factor mean difference between classes is smaller (underestimation).

**Case 4. Negative pairing: A latent class with higher factor loadings associated with lower factor means**

This is the case in which both factor loadings and factor means are noninvariant ($\boldsymbol{\Lambda}_{c}$ and $\boldsymbol{\kappa}_{c}$). We use the same setting in Case 2 except that

$$\boldsymbol{\kappa}_{1}=\left[ \begin{aligned} 0 \\ 1 \end{aligned} \right], \boldsymbol{\kappa}_{2}=\left[ \begin{aligned} 1.4 \\ 1.4 \end{aligned} \right].$$

Thus, the expected means of six observed variables for each class are

$$\boldsymbol{\mu}_{1}=\left[ \begin{aligned} 0 \\ 0 \\ 1 \\ 1 \\ 2 \\ 2 \end{aligned} \right], \boldsymbol{\mu}_{2}=\left[ \begin{aligned} 1.4 \\ 0.84 \\ 2.8 \\ 1.68 \\ 4.2 \\ 2.52 \end{aligned} \right].$$

When the noninvariance in factor loadings is correctly modeled, the estimated factor means are:

$$\boldsymbol{\kappa}_{1}\boldsymbol{=}\boldsymbol{\Gamma}^{-1}{\boldsymbol{\Lambda}_{\boldsymbol{1}}}^{-1}\left( \boldsymbol{\mu}_{1}-\boldsymbol{\nu} \right)=\left[ \begin{matrix} 1 & 0 \\ 1 & 1 \\ 1 & 2 \end{matrix} \right]^{\boldsymbol{-1}}\boldsymbol{*}\left[ \begin{aligned} \begin{matrix} 1 & 0 & 0 \\ 1 & 0 & 0 \\ 0 & 1 & 0 \end{matrix} \\ \begin{matrix} 0 & 1 & 0 \\ 0 & 0 & 1 \\ 0 & 0 & 1 \end{matrix} \end{aligned} \right]^{\boldsymbol{-1}}\boldsymbol{*}\left( \left[ \begin{aligned} 0 \\ 0 \\ 1 \\ 1 \\ 2 \\ 2 \end{aligned} \right]\boldsymbol{-}\left[ \begin{aligned} 0 \\ 0 \\ 0 \\ 0 \\ 0 \\ 0 \end{aligned} \right] \right)\boldsymbol{=}\left[ \begin{aligned} 0 \\ 1 \end{aligned} \right],$$

$$\boldsymbol{\kappa}_{2}\boldsymbol{=}\boldsymbol{\Gamma}^{-1}{\boldsymbol{\Lambda}_{\boldsymbol{2}}}^{-1}\left( \boldsymbol{\mu}_{2}-\boldsymbol{\nu} \right)=\left[ \begin{matrix} 1 & 0 \\ 1 & 1 \\ 1 & 2 \end{matrix} \right]^{\boldsymbol{-1}}\boldsymbol{*}\left[ \begin{aligned} 1 \\ 0.6 \\ 0 \\ 0 \\ 0 \\ 0 \end{aligned} \begin{aligned} 0 \\ 0 \\ 1 \\ 0.6 \\ 0 \\ 0 \end{aligned} \begin{aligned} 0 \\ 0 \\ 0 \\ 0 \\ 1 \\ 0.6 \end{aligned} \right]^{\boldsymbol{-1}}\boldsymbol{*}\left( \left[ \begin{aligned} 1.4 \\ 0.84 \\ 2.8 \\ 1.68 \\ 4.2 \\ 2.52 \end{aligned} \right]\boldsymbol{-}\left[ \begin{aligned} 0 \\ 0 \\ 0 \\ 0 \\ 0 \\ 0 \end{aligned} \right] \right)\boldsymbol{=}\left[ \begin{aligned} 1.4 \\ 1.4 \end{aligned} \right].$$

When the noninvariance is assumed and one set of factor loadings are applied to both classes ($\boldsymbol{\Lambda}_{\boldsymbol{1}}^{'}=\boldsymbol{\Lambda}_{\boldsymbol{2}}^{'}=\left[ 1 0.8 1 0.8 1 0.8 \right]$), the estimated factor means are:

$$\boldsymbol{\kappa}_{1}\boldsymbol{=}\boldsymbol{\Gamma}^{-1}\boldsymbol{\Lambda}^{-1}\left( \boldsymbol{\mu}_{1}-\boldsymbol{\nu} \right)=\left[ \begin{matrix} 1 & 0 \\ 1 & 1 \\ 1 & 2 \end{matrix} \right]^{\boldsymbol{-1}}\boldsymbol{*}\left[ \begin{aligned} 1 \\ 0.8 \\ 0 \\ 0 \\ 0 \\ 0 \end{aligned} \begin{aligned} 0 \\ 0 \\ 1 \\ 0.8 \\ 0 \\ 0 \end{aligned} \begin{aligned} 0 \\ 0 \\ 0 \\ 0 \\ 1 \\ 0.8 \end{aligned} \right]^{\boldsymbol{-1}}\boldsymbol{*}\left( \left[ \begin{aligned} 0 \\ 0 \\ 1 \\ 1 \\ 2 \\ 2 \end{aligned} \right]\boldsymbol{-}\left[ \begin{aligned} 0 \\ 0 \\ 0 \\ 0 \\ 0 \\ 0 \end{aligned} \right] \right)\boldsymbol{=}\left[ \begin{aligned} 0 \\ 1.1 \end{aligned} \right],$$

$$\boldsymbol{\kappa}_{2}\boldsymbol{=}\boldsymbol{\Gamma}^{-1}\boldsymbol{\Lambda}^{-1}\left( \boldsymbol{\mu}_{2}-\boldsymbol{\nu} \right)=\left[ \begin{matrix} 1 & 0 \\ 1 & 1 \\ 1 & 2 \end{matrix} \right]^{\boldsymbol{-1}}\boldsymbol{*}\left[ \begin{aligned} 1 \\ 0.8 \\ 0 \\ 0 \\ 0 \\ 0 \end{aligned} \begin{aligned} 0 \\ 0 \\ 1 \\ 0.8 \\ 0 \\ 0 \end{aligned} \begin{aligned} 0 \\ 0 \\ 0 \\ 0 \\ 1 \\ 0.8 \end{aligned} \right]^{\boldsymbol{-1}}\boldsymbol{*}\left( \left[ \begin{aligned} 1.4 \\ 0.84 \\ 2.8 \\ 1.68 \\ 4.2 \\ 2.52 \end{aligned} \right]\boldsymbol{-}\left[ \begin{aligned} 0 \\ 0 \\ 0 \\ 0 \\ 0 \\ 0 \end{aligned} \right] \right)\boldsymbol{=}\left[ \begin{aligned} 1.3 \\ 1.3 \end{aligned} \right].$$

The slope factor mean of a class with lower factor means is overestimated whereas that of a class with higher factor means are underestimated, which leads to the underestimation of the mean difference between classes. In this case the intercept factor means are also affected by the ignored noninvariance.

When a latent class with higher factor loadings is paired with higher factor means (positive pairing), the slope factor mean of this class will be overestimated whereas that of the class with lower factor means will be underestimated, which makes the slope factor mean difference between classes is larger (overestimation).
